# The Bioinformatics Analysis of Comparative Genomics of *Mycobacterium tuberculosis* Complex (MTBC) Provides Insight into Dissimilarities between Intraspecific Groups Differing in Host Association, Virulence, and Epitope Diversity

**DOI:** 10.3389/fcimb.2017.00088

**Published:** 2017-03-21

**Authors:** Xinmiao Jia, Li Yang, Mengxing Dong, Suting Chen, Lingna Lv, Dandan Cao, Jing Fu, Tingting Yang, Ju Zhang, Xiangli Zhang, Yuanyuan Shang, Guirong Wang, Yongjie Sheng, Hairong Huang, Fei Chen

**Affiliations:** ^1^CAS Key Laboratory of Genome Sciences & Information, Beijing Institute of Genomics, Chinese Academy of SciencesBeijing, China; ^2^College of Life Sciences, University of Chinese Academy of SciencesBeijing, China; ^3^National Clinical Laboratory on Tuberculosis, Beijing Key Laboratory on Drug-resistant Tuberculosis Research, Beijing Chest Hospital, Capital Medical University, Beijing Tuberculosis and Thoracic Tumor InstituteBeijing, China; ^4^Key Laboratory for Molecular Enzymology and Engineering of Ministry of Education, Jilin UniversityChangchun, China; ^5^Sino-Danish College, University of Chinese Academy of SciencesBeijing, China; ^6^Collaborative Innovation Center for Genetics and DevelopmentShanghai, China

**Keywords:** *Mycobacterium tuberculosis* complex (MTBC), tuberculosis (TB), host association, virulence, epitope, comparative genomics, pathogenicity, PacBio

## Abstract

Tuberculosis now exceeds HIV as the top infectious disease cause of mortality, and is caused by the *Mycobacterium tuberculosis* complex (MTBC). MTBC strains have highly conserved genome sequences (similarity >99%) but dramatically different phenotypes. To analyze the relationship between genotype and phenotype, we conducted the comparative genomic analysis on 12 MTBC strains representing different lineages (i.e., *Mycobacterium bovis*; *M. bovis* BCG; *M. microti*; *M. africanum*; *M. tuberculosis* H37Rv; *M. tuberculosis* H37Ra, and six *M. tuberculosis* clinical isolates). The analysis focused on the three aspects of pathogenicity: host association, virulence, and epitope variations. Host association analysis indicated that eight *mce3* genes, two enoyl-CoA hydratases, and five PE/PPE family genes were present only in human isolates; these may have roles in host-pathogen interactions. There were 15 SNPs found on virulence factors (including five SNPs in three ESX secretion proteins) only in the Beijing strains, which might be related to their more virulent phenotype. A comparison between the virulent H37Rv and non-virulent H37Ra strains revealed three SNPs that were likely associated with the virulence attenuation of H37Ra: S219L (PhoP), A219E (MazG) and a newly identified I228M (EspK). Additionally, a comparison of animal-associated MTBC strains showed that the deletion of the first four genes (i.e., *pe35, ppe68, esxB, esxA*), rather than all eight genes of RD1, might play a central role in the virulence attenuation of animal isolates. Finally, by comparing epitopes among MTBC strains, we found that four epitopes were lost only in the Beijing strains; this may render them better capable of evading the human immune system, leading to enhanced virulence. Overall, our comparative genomic analysis of MTBC strains reveals the relationship between the highly conserved genotypes and the diverse phenotypes of MTBC, provides insight into pathogenic mechanisms, and facilitates the development of potential molecular targets for the prevention and treatment of tuberculosis.

## Introduction

According to a 2016 World Health Organization (WHO) report, tuberculosis (TB) has surpassed HIV as the infectious disease causing the highest number of mortalities, with an estimated 1.8 million deaths and 10.4 million new TB cases worldwide in 2015 (World Health Organization, 2016). The primary cause of TB, *Mycobacterium tuberculosis* (Mtb), belongs to the *M. tuberculosis* complex (MTBC). MTBC is a genetically related group of *Mycobacterium* species that can cause TB in humans or other organisms (Galagan, 2014). In general, MTBC can be classified into eight main lineages (Lineage 1-8, L1-8): L1 (The Philippines and Indian Ocean), L2 (East Asia), L3 (India and East Africa), L4 (Europe and Americas), L5 (West Africa 1), L6 (West Africa 2), L7 (Ethiopia), and L8 (animal-adapted isolates). Among these, L1, L2, L3, L4, and L7 comprise Mtb. MTBC can also be divided into ancient (L1, L5-8) and modern strains (L2-4) according to the presence or absence of an *M. tuberculosis* specific deletion (TbD1; Brosch et al., 2002; Galagan, 2014).

After the first published genome of *M. tuberculosis* in 1998 (Cole et al., 1998), there were many large-scale genomic studies involving many MTBC strains (>3,500) (http://www.ncbi.nlm.nih.gov/genome/genomes). Analysis of worldwide sequencing data shows that MTBC members share more than 99% identity at the nucleotide level (Brosch et al., 2000; Rodriguez-Campos et al., 2014). Also, large genomic rearrangements are infrequent in MTBC strains (Galagan, 2014).

Although MTBC members have almost identical genome sequences/genotypes (similarity >99%) (Brosch et al., 2000), they exhibit diverse pathogenic phenotypes, which are thought to be the result of long-term co-evolution of MTBC strains with a diverse group of humans and animals (Comas et al., 2013; Galagan, 2014). Long-term geographical isolation gave rise to the accumulation of “genotype isolation,” thus resulting in different phenotypes (Ghebremichael et al., 2010).

Firstly, the MTBC members have diverse host associations. The L1 to L7 lineages mainly infect humans and rarely infect animals. Among these, the L5 and L6 lineages (*M. africanum* I and II) only cause human TB in West Africa (Bentley et al., 2012; Winglee et al., 2016), and the L7 lineage has only been reported in Ethiopia or in Ethiopian emigrants located in Djibouti (Firdessa et al., 2013). The other human-associated MTBC lineages (L1-4) also exhibit relatively high levels of distinct geographic distributions (Galagan, 2014). These findings suggest that the MTBC lineages have coevolved with diverse hominid ancestors since ancient times, which has lead to their diverse adaptations to specific human populations (Gagneux et al., 2006; Comas et al., 2013; Galagan, 2014). In addition, the L8 lineage (consisting of different animal-adapted MTBC isolates: *M. bovis, M. bovis* BCG, and *M. microti*, among others) has diverse animal-host adaptations, likely for the same reason (Brosch et al., 2002; Rodriguez-Campos et al., 2014). *M. bovis* rarely causes TB in humans but it is highly virulent for cattle (Brosch et al., 2000). Similarly, *M. microti* has been reported to mainly infect rodents, such as voles, and rarely infects humans (Brosch et al., 2000).

Secondly, MTBC strains have different levels of virulence [i.e., the degree of pathogenicity or the ability of the organism to invade the tissues of the host (Pirofski and Casadevall, 2012)]. The modern strains (L2-L4) are more virulent to humans than the ancient strains (L1; L5-8), and are responsible for the vast majority of today's TB cases (Brosch et al., 2002; Gagneux, 2012). Among these, the Beijing sub-lineage of the L2 lineage appears to be more virulent than the other modern isolates due to its enhanced resistance and adaptation (Ida et al., 2010).

The virulence of MTBC strains is not set in stone. For example, *M. tuberculosis* H37Ra, isolated by William Steenken in 1935 (Steenken and Gardner, 1946), is usually regarded as the non-virulent counterpart of the virulent H37Rv strain. Historically, both *M. tuberculosis* H37Ra and H37Rv are derived from the same parent strain (H37). H37Ra and H37Rv are the most common standard strains of *M. tuberculosis* in laboratories. Another famous example is *M. bovis* BCG, which was isolated from virulent *M. bovis* after 239 passages (Liu et al., 2009); this is the most widely used TB vaccine in the world.

Thirdly, some studies have shown a different immune response to different MTBC strains (Gagneux, 2012). It is reported that hypervirulent strains induce lower immune responses than low-pathogenic strains (Portevin et al., 2011). The distinct capacity of virulent strains to remain sequestered in host macrophages seems to be a reason for their ability to evade immune responses (Chen et al., 2006; Grace and Ernst, 2016).

In recent years, some genomic studies concerning the relationship between the genotype and phenotype of MTBC strains have been reported. A number of mycobacterial virulence genes (i.e., virulence factors) have been identified; most of these encode for cell surface proteins or lipid and fatty acid metabolism proteins (Forrellad et al., 2013). Some comparative genomic analyses between the virulent H37Rv and the non-virulent H37Ra strains revealed that the mutation of Ser219 into leucine of the PhoP virulence factor might result in the attenuation of virulence in H37Ra (Ryndak et al., 2008; Zheng et al., 2008). In addition, it is also known that the deletion of region of difference 1 (RD1) in the genome of *M. bovis* BCG led to its attenuation (based on a comparison with *M. bovis*) (Pym et al., 2002; Lewis et al., 2003).

To date, many of the comparative genomic studies only have been focused on the comparison between virulent and non-virulent MTBC strains; thus, comparative genomic studies covering the host association, virulence, and epitope variations are still lacking. This is mainly due to the fact that, on one hand, precise comparisons and analyses of almost identical MTBC genome sequences (similarity >99%) are needed to obtain precise and complete MTBC genome sequences; meanwhile, on the other hand, it is very difficult to obtain complete MTBC genome sequences using second generation sequencing technologies, such as the Illumina Hiseq platform, due to the high GC content and repetitive sequences of the PE/PPE multi-gene family.

Herein, the 12 MTBC genomes, we finished using Pacbio single-molecule real-time (SMRT) technology (Zhu et al., 2016), provided a groundwork for analyzing the relationship between the genotype and phenotype of MTBC. To obtain a precise analysis, we re-sequenced the genomes of 12 MTBC strains using an Illumina Hiseq. Based on the 12 precise and complete MTBC genomes, we performed a comparative genomic analysis looking at three aspects of pathogenicity: host association, virulence, and epitope diversity.

## Materials and methods

### Isolates and genotyping

The 12 MTBC isolates, which included six reference strains and six clinical isolates, have been previously described (Table S1; Zhu et al., 2016). Six standard reference strains were obtained from American Type Culture Collection (ATCC) specifically for the purpose of genome sequencing. The strains were grown in either Lowenstein–Jenden media or Middlebrook 7H10 media supplemented with 10% OADC (Oleic Albumin Dextrose Catalase, Becton Dickinson), glycerol, and 0.05% Tween 80. We used the VNTR-15 scheme as described in MIRU-VNTRplus (http://www.miru-vntrplus.org/) for genotyping, which uses the following markers: Mtub04; ETRC; MIRU04; MIRU40; MIRU10; MIRU16; Mtub21; QUB11b; ETRA; Mtub30; MIRU26; MIRU31; Mtub39; QUB26, and QUB4156. Each MIRU-VNTR locus was individually amplified, and electrophoresis of products on agarose gels was conducted as previously described (Fabre et al., 2004). The copy number at each locus was calculated in BioNumerics. A large sequence polymorphism (LSP) (pks15/1) was used to differentiate between the clinical TB strains using the primers shown in Table S2. A deletion of a 7-bp region in the polyketide synthase gene pks15/1 is present in the Euro-American lineage of *M. tuberculosis*. Whole genome SNP typing was also done using MEGA 6.06 (maximum likelihood method) as previously described (Tamura et al., 2013; Zhu et al., 2016). The results of this analysis are consistent with those of the VNTR analysis (Figure S1).

The 12 MTBC strains covered five lineages. There were two clinical L2 strains (Mtb 2242 and 2279), one clinical L3 strain (Mtb 26105), five L4 strains (three clinical strains-Mtb 22115, Mtb 22103, and Mtb 37004 and two reference strains-Mtb F1 and Mtb F28), one L6 reference strain (*M. africanum* 25), and three L8 reference strains (*M. microti* 12, *M. bovis* 30, and *M. bovis* BCG 26). Of the 12 strains, Mtb 2242, Mtb 2279, Mtb 26105, Mtb 22115, Mtb 22103, Mtb 37004, Mtb F1, Mtb F28, and *M. africanum* 25 are human isolates; meanwhile, *M. microti* 12, *M. bovis* 30, and *M. bovis* BCG 26 are animal isolates. In addition, the 12 MTBC isolates contained two homologous pairs of virulent/non-virulent strains: virulent *M. tuberculosis* H37Rv (Mtb F1: ATCC27294) and its non-virulent counterpart H37Ra (Mtb F28: ATCC25177), and virulent *M. bovis* (*M. bovis* 30: ATCC19210) and its non-virulent counterpart *M. bovis* BCG (*M. bovis* BCG 26: ATCC35735).

### Genomic DNA extraction, sequencing, correction and re-annotation

Genomic DNA from the 12 MTBC strains was extracted using a TIANamp Bacteria Genomic DNA Kit (Tiangen BiotechCo. Ltd., Beijing, China), and was sequenced using PacBio Single-Molecule Real-Time (SMRT) Technology as previously described (Zhu et al., 2016).

To correct the polymer errors produced during PacBio sequencing, we re-sequenced the 12 isolates using next-generation sequencing. The genomes of the 12 isolates were shotgun sequenced using an Illumina Genome Analyzer 2X. Paired-end libraries were prepared from 5 ug of isolated genomic DNA using the TruSeq DNA sample prep kit A (Illumina Inc., San Diego) according to the manufacturer's instructions. Genomic paired-end libraries were sequenced with a read length of 2 × 150 nucleotides using an Illumina GAIIx instrument according to the manufacturer's instructions. Image analysis and base calling were done in the standard Illumina pipeline. The raw Illumina sequencing reads were trimmed at a threshold of 0.01 (Phred score of 20). Filtered reads were mapped onto the genome sequences, which were assembled by the Hierarchical Genome Assembly Process (HGAP.3) algorithm in SMRT Portal (version 2.2.0) using BWA version 0.5.9 (Li and Durbin, 2010), and converted to sorted BAM format using SAMtools v0.1.9 (Li et al., 2009). The coverage ranged between 157× and 394× with an average of 255×. Pilon v1.13 (Walker et al., 2014) was then used to polish the genome sequences using these alignments, which resulted in a total of 9,493 insertions and 133 deletions. All the raw Illumina sequencing reads have also been deposited in the National Center for Biotechnology Information (NCBI) Sequence Read Archive (SRA) database (SRP064893) and the Genome Sequence Archive (GSA) of the BIG Data Center (BIGD) (PRJCA000307).

All 12 genome sequences were re-annotated with the Rapid Annotation tool in the Subsystem Technology (RAST) pipeline, which is a fully automated annotation engine for complete or draft archaeal and bacterial genomes (Aziz et al., 2008).

### Genome structure and identification of specific genes and single-nucleotide polymorphisms (SNPs)

The average nucleotide identity (ANI) was calculated through ANI on EzGenome (http://www.ezbiocloud.net/tools/ani). Multiple alignments of genomic sequences were performed using the Mauve multiple alignment software with the progressive alignment option (Darling et al., 2010). The output file produced by Mauve was parsed using a custom Perl script (Supplementary Data) to retrieve multiple aligned sequences for SNP loci. SNPs called in repetitive regions of the genome, which were defined as exact repetitive sequences ≥25 bp in length, and were identified using Repeat-Masker (Tarailo-Graovac and Chen, 2009), were excluded.

Gene sequences were downloaded from the RAST server after annotation. To identify specific genes, ortholog clustering was performed using orthoMCL (Li et al., 2003). Each cluster is a homology group. If the genes in a homology group covered all species in a group but no strains in other groups, we considered the genes in this group to be group specific genes.

### Epitopes, virulence factors and PE/PPE gene family distribution

Epitopes were obtained from the Immune Epitope Database (IEDB; Vita et al., 2015), and those that had been positively experimentally identified were selected and renamed. Only epitopes with 100% identical BLAT matches were considered to be the same epitope. The epitope was classified as an absence epitope if it was located in an absence gene or in a deleted/mutated region of a non-absence gene. Epitopes with the same distribution pattern were clustered into one epitope_cluster. Furthermore, BLASTn was used to search for differences between 12 genomes in terms of single nucleotide variations, insertions, and deletions in corresponding antigen genes.

Virulence genes were downloaded from the Virulence Factor Database (VFDB; Chen et al., 2005; Table S3). All of the PE/PPE genes analyzed in this study (Table S4) were based on a search of the *M. tuberculosis* H37Rv genes (NC_000962) from the NCBI gene database (www.ncbi.nlm.nih.gov/gene). FASTA sequences of these genes were used to search for corresponding genes in the 12 genomes using BLAST (50% coverage and 90% identity thresholds).

## Results and discussion

### Conserved genomes (genotypes) and diverse phenotypes of 12 MTBC strains

The MTBC genomes obtained by SMRT sequencing (Zhu et al., 2016) were re-sequenced to correct the homo-polymer errors using Illumina sequencing. Based on an average of 5,394,126 paired-end reads (255X coverage) (SRP064893), we resolved a total of 9,493 insertions and 133 deletions compared with the original genomes (Table S5). The precise genomes provided general information (Table 1), including the size of the genomes (4.34–4.43 Mb), the number of predicted protein-coding genes (~4,400), and the gene length (~900bp). Importantly, the ANI and SNP analyses showed that the MTBC genomes were highly conserved, as the maximum number of SNPs and the minimum ANI were 2,356 and 99.75%, respectively (Table 1). Pan-genomic analysis was further implemented and used to identified 3,761 core gene clusters (Figure S2A), which accounted for ~87% of all genes. This also demonstrates that MTBC genomes are highly conserved.

**Table 1:** **Conserved genomes (genotypes) and diverse phenotypes of 12 MTBC strains**.

	**ATCC No./Lineage**	**Strain No**.	**Species**	**Host**	**Completed genome size (bp)**	**ANI^a^ (%)**	**Coding gene number**	**Core gene number**	**SNPs^b^**	**Virulence factor**	**Epitope**
Modern strains	27294/L4	F1	*M. tuberculosis* H37Rv	Human	4,429,062	99.9845	4,400	3,834	110	253	2,208
	25177/L4	F28	*M. tuberculosis* H37Ra		4,421,992	99.9893	4,366	3,837	92	252	2,205
	L4	22,115	*M. tuberculosis*		4,402,103	99.9112	4,356	3,827	840	253	2,201
	L4	37,004	*M. tuberculosis*		4,417,474	99.8955	4,375	3,820	855	252	2,189
	L4	22,103	*M. tuberculosis*		4,399,638	99.8498	4,345	3,809	1,033	253	2,175
	L3	26,105	*M. tuberculosis*		4,426,728	99.8796	4,393	3,833	1,504	251	2,261
	L2	2,242	*M. tuberculosis*		4,420,756	99.8322	4,428	3,839	1,434	251	2,128
	L2	2,279	*M. tuberculosis*		4,406,429	99.8436	4,400	3,839	1,514	252	2,181
Ancient strains	35711/L6	25	*M. africanum*	Human (tropical Africa)	4,388,515	99.7731	4,385	3,819	2,336	246	2,135
	19422/L8	12	*M. microti*	Voles and rodents	4,370,890	99.7712	4,360	3,815	2,158	232	1,756
	19210/L8	30	*M. bovis*	Wide range of mammals especially cattle	4,336,684	99.7680	4,312	3,804	2,345	238	2,137
	35735/L8	26	*M. bovis* BCG		4,353,641	99.7488	4,348	3,832	2,356	236	1,706

a ANI: Average Nucleotide Identity;

b *Reference genome: H37Rv (NC_000962)*.

**Figure 1:**
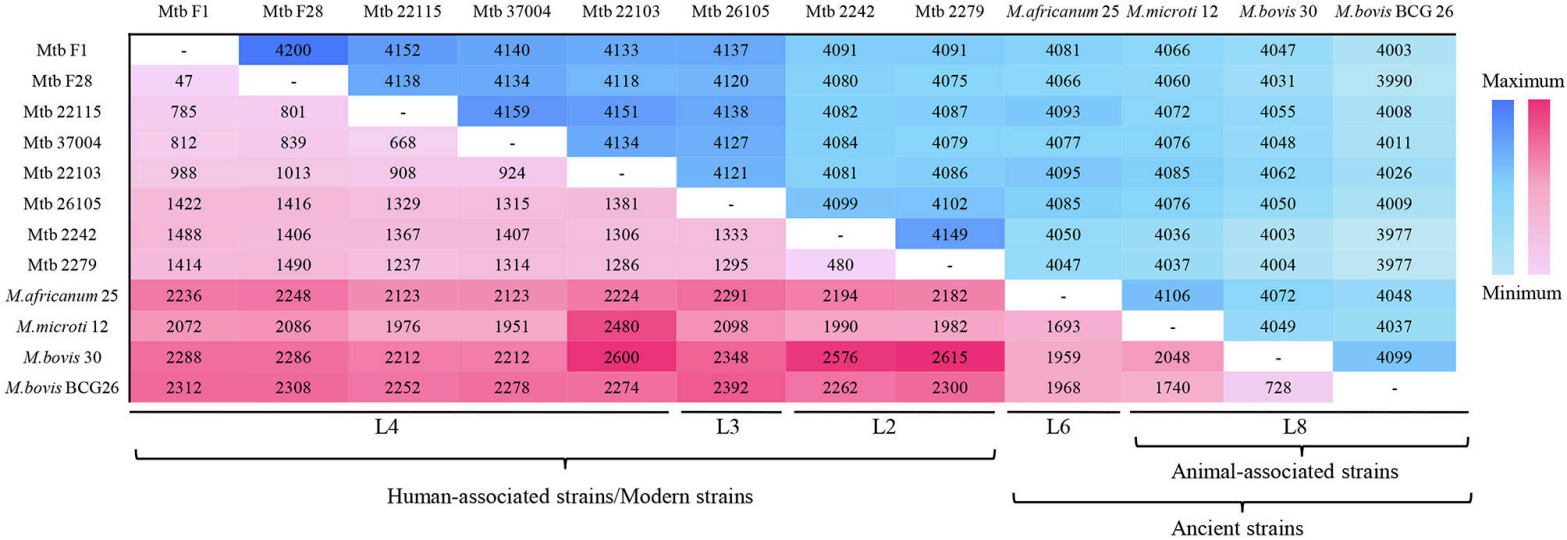
**Pair-wise comparisons of SNPs and orthologous genes in 12 MTBC strains**. The plum red region represents the SNP number, and the blue region indicates the orthologous gene number. The colors darken with increases in number.

The comparative genomic analysis also showed some subtle differences amongst the 12 MTBC genomes: the five L4 strains had fewer SNPs (<1,000) because the reference genome (NC_000962) belonged to the L4 lineage; the three animal-associated MTBC strains possessed more SNPs (>2,000), and the two L2 and one L3 strains all had ~1,500 SNPs (Table 1). The number of SNPs reflected the genetic distance from the reference L4 strain (Takezaki and Nei, 1996). To lower the influence of the reference, pair-wise comparisons of SNPs were conducted (Figure 1). This comparison proved that the differences in SNPs between the animal- and human-associated strains were much greater than those between intra-lineage strains. On the other hand, the analyses for the pan-genomic and pair-wise comparisons of orthologous genes (Figure S2 and Figure 1, respectively) indicated some strain-specific and lineage-specific genes.

It is well-known that genotype is a major factor influencing phenotype. Thus, the pathogenic phenotype differences amongst MTBC lineages can also be attributed to the small number of genomic variations, including the specific genes and SNPs described above. In the following sections, we investigate the correlation between these genetic variations and three essential phenotypic features (host association, virulence, and epitope variation).

### Host association analysis

In an attempt to reveal the genetic basis for host association, we performed a comparative genomic study between the strains isolated from human (i.e., Mtb 2242, Mtb 2279, Mtb 26105, Mtb 22115, Mtb 22103, Mtb 37004, Mtb F1, and Mtb F28) and animal (i.e., *M. microti* 12, *M. bovis* 30, and *M.bovis* BCG 26). The average ANI was 99.76% (99.71–99.80%; Table S6), and no large genome structure variation was observed. Minor differences in genome sequences between these two groups of strains might explain the differences in host association. We found 29 genes specific to human isolates (Table 2), 16 genes specific to animal isolates (Table S7), and 579 SNPs (Table S8).

**Table 2:** **Human-associated strain specific genes^a^ [Reference genome: H37Rv (NC_000962)]**.

**Gene**	**Synonym**	**Product length**	**COG**	**Annotation**	**RD No**.
–	Rv0221	469	COG4908R	Diacyglycerol O-acyltransferase	RD10
echA1	Rv0222	262	COG1024I	Enoyl-CoA hydratase EchA1	RD10
PE_PGRS5	Rv0297	591	–	PE-PGRS family protein PE_PGRS5	/
PPE7	Rv0354c	141	–	PPE family protein PPE7	/
galTb	Rv0619	181	COG1085G	Galactose-1-phosphate uridylyltransferase GalTb	/
–	Rv1503c	182	COG0399M	TDP-4-oxo-6-deoxy-D-glucose aminotransferase	/
PE_PGRS31	Rv1768	618	COG5164	PE-PGRS family protein PE_PGRS31	RD14
yrbE3A	Rv1964	265	COG0767Q	Integral membrane protein	RD7
yrbE3B	Rv1965	271	COG0767Q	Integral membrane protein	RD7
mce3A	Rv1966	425	COG1463Q	Mce family protein Mce3A	RD7
mce3B	Rv1967	342	COG1463Q	Mce family protein Mce3B	RD7
mce3C	Rv1968	410	COG1463Q	Mce family protein Mce3C	RD7
mce3D	Rv1969	423	COG1463Q	Mce family protein Mce3D	RD7
lprM	Rv1970	377	COG1463Q	Mce family lipoprotein LprM	RD7
mce3F	Rv1971	437	COG1463Q	Mce family protein Mce3F	RD7
–	Rv1972	191	–	Mce associated membrane protein	RD7
–	Rv1973	160	–	Mce associated membrane protein	RD7
–	Rv1974	125	–	Membrane protein	RD7
–	Rv1975	221	COG2340S	Hypothetical protein	RD7
–	Rv1976c	135	–	Hypothetical protein	RD7
–	Rv1977	348	COG0501O	Hypothetical protein	RD7
–	Rv2073c	249	COG0300R	Oxidoreductase	RD9
–	Rv2074	137	–	Pyridoxamine 5'-phosphate oxidase	RD9
–	Rv2227	233	COG3826S	Hypothetical protein	/
echA18	Rv3374	82	COG1024I	Enoyl-CoA hydratase	/
ephA	Rv3617	322	COG0596R	Epoxide hydrolase EphA	RD8
–	Rv3618	395	COG2141C	Monooxygenase	RD8
PPE65	Rv3621c	413	COG5651N	PPE family protein PPE65	RD8
PE32	Rv3622c	99	–	PE family protein PE32	RD8

a *Eight mce3 family genes, two enoyl-CoA hydratases and five PE/PPE family genes are highlighted as red, green, and blue letters*.

Interestingly, in the 29 genes specific to human isolates, 14 in region of difference 7 (RD7; Table 2) were clustered together (Figure S3). Among these, eight mammalian cell entry 3 (mce3) family genes appear to play roles in human host association of MTBC strains, since the Mce protein is an important protein family for entry and survival of bacteria in the host (Harboe et al., 2002; Ahmad et al., 2004). They are located on the cell surface and reside in the same *mce3* operon of *M. tuberculosis* (Harboe et al., 2002). Additionally, the two genes upstream (*yrbEA* and *yrbEB*) from the eight *mce3* genes and the four genes downstream (*Rv1974, Rv1975, Rv1976c*, and *Rv1977*) from the eight *mce3* genes encode integral membrane proteins and signal sequences, respectively (Harboe et al., 2002). Their absence in animal isolates might be related to host association.

It is also worth noting that two enoyl-coenzyme A (CoA) hydratases (EchA1 and EchA18) were only present in human isolates (Table 2); these hydratases are key enzymes in the fatty acid β-oxidation pathway. Through β-oxidative reactions, host-cell lipids can be degraded and provide precursors for many metabolic processes, such as cell-wall synthesis (Cole et al., 1998). Therefore, the absence of enoyl-CoA hydratase in animal-associated MTBC strains might impact fatty acid metabolism and cell-wall synthesis in host cells.

In addition, five PE/PPE family genes (i.e., *ppe7, ppe65, pe32, pe_pgrs5*, and *pe_pgrs31*) were found to exist only in the human isolates (Table 2). Most of the PE/PPE proteins were localized or secreted to the cell surface; these proteins have been implicated in mycobacterial antigenic variation and host immune evasion (Akhter et al., 2012). Thus, the absence of these five PE/PPE proteins in animal isolates might lead to differences in host association.

Of the 29 genes specific to human isolates, *ppe65, echA1, ephA*, and *Rv1977* are known antigen genes (Vita et al., 2015). The loss of these four genes might lead to changes in the immune response of animal isolates (discussed in the epitope section).

Five hundred and Seventy nine SNPs were identified between the human- and animal-isolates; 315 non-synonymous SNPs were distributed on 287 genes (Table S8A). Among them, 26 SNPs were on virulence factors and seven SNPs were located on antigen genes (Table S8B). It is likely that they influence protein function and further affect mycobacterial host association.

### Genomic analysis of virulence differences

Diverse MTBC lineages have different degrees of virulence (Brosch et al., 2002; Ida et al., 2010; Gagneux, 2012) even though their genomes are highly conserved. In this study, we searched for virulence factors in the 12 MTBC genomes by performing a BLAST search against 257 virulence factors collected from the VFDB (Chen et al., 2005; Table S3). Among these, 86 are experimentally validated (Table S3A), while the others are putative ones (Table S3B). The results showed that more than 80% (216: 56 experimentally validated and 160 putative) of the virulence factors were existed and conserved in all 12 strains (Figure S2B). Also, we found that the number of virulence factors in the four ancient strains was less than in the eight modern strains, which is in agreement with the more virulent phenotype of modern strains (Gagneux, 2012).

It is well known that Beijing sub-lineage strains in the L2 lineage are more virulent than the other modern isolates (Ida et al., 2010). There was no appreciable difference between Beijing sub-lineage strains (Mtb 2242 and Mtb 2279) and other modern strains (Mtb F1, Mtb F28, Mtb 22115, Mtb 37004, Mtb 22103, and Mtb 26105) in regards to the number and species of virulence factors (Table 1). We further investigated the SNPs between the Beijing and other modern strains, and 18 non-synonymous SNPs were identified (Table 3). They were then checked by performing a BLAST search against ten other Beijing strains with complete genomes from NCBI GenBank (Table S9A). 15 SNPs on 13 virulence factors (five validated and eight putative) were confirmed to exist in the 12 Beijing strains. Most noteworthy were the five SNPs located on the ESX secretion systems, which are very important for *M. tuberculosis* pathogenesis (Simeone et al., 2015).

**Table 3:** **The non-synonymous mutations between Beijing strains and other MTBC strains on virulence factors**.

**Gene**	**Synonym**	**Position**	**SNPs**	**Annotation**
mce1D	Rv0172	563 (188)	T->C (I->T)	Mce family protein Mce1D
eccD3^a^	Rv0290	227 (76)	G->A (S->N)	ESX-3 secretion system protein EccD
eccD3	Rv0290	283 (95)	G->A (A->T)	ESX-3 secretion system protein EccD
mce2F	Rv0594	1,295 (432)	A->G (N->S)	Mce family protein Mce2F
mmpL10	Rv1183	1,222 (408)	A->G (T->A)	Transmembrane transport protein MmpL10
plcC	Rv2349c	1,081 (361)	G->T (G->C)	Phospholipase C
plcA	Rv2351c	1,336 (446)	A->G (T->A)	Membrane-associated phospholipase A
mbtB	Rv2383c	2,020 (674)	G->C (V->L)	Phenyloxazoline synthase
ppsA	Rv2931	3,581 (1194)	T->G (L->R)	Phthiocerol synthesis polyketide synthase type I PpsA
Mas	Rv2940c	6,013 (2005)	A->C (T->P)	Multifunctional mycocerosic acid synthase
–	Rv2952	526 (176)	G->A (G->R)	Phthiotriol/phenolphthiotriol dimycocerosates methyltransferase
kefB	Rv3236c	304 (102)	A->G (T->A)	Integral membrane transport protein
lipF	Rv3487c	697 (233)	C->T (R->C)	Carboxylesterase LipF
papA2	Rv3820c	1,397 (466)	C->T (P->L)	Trehalose-2-sulfate acyltransferase
fadD23	Rv3826	1,264 (422)	G->C (E->Q)	Long-chain-fatty-acid–CoA ligase FadD23
espK	Rv3879c	130 (44)	G->A (D->N)	ESX-1 secretion-associated protein EspK
espK	Rv3879c	1,979 (660)	A->C (E->A)	ESX-1 secretion-associated protein EspK
eccC2	Rv3894c	1,949 (650)	A->G (D->G)	ESX-2 type VII secretion system protein EccC

a *SNPs in red are the ones that are further validated in other ten Beijing strains*.

The two homologous pairs of virulent/non-virulent strains (i.e., H37Rv vs. H37Ra and *M. bovis* vs. *M. bovis* BCG) are well suited for virulence genetic analysis because they have the same origins (Steenken and Gardner, 1946; Liu et al., 2009). Thus, we conducted a comparative genomic analysis between virulent and non-virulent strains at the whole genome level (not limited to virulence factors).

#### H37Rv vs. H37Ra

H37Ra and H37Rv show very different virulence even though they share an origin. To investigate the relationship between the phenotypic differences and genetic variations, we implemented a comparative genomic study between the five H37Rv (including four online complete genomes from NCBI GenBank and Mtb H37Rv F1 genome) and two H37Ra genomes (including one online complete genome from NCBI GenBank and Mtb H37Ra F28 genome; Table S9B). ANI analysis revealed 99.98% identity between the H37Rv and H37Ra strains. No large genome structure variation was found. Four specific genes in all H37Ra strains and five missense SNPs were identified.

The four H37Ra specific genes were clustered together and located in RvD2 (H37Rv-related deletion 2) with two IS6110 transposase genes on both ends (Figure S4). Further, comparison with the other three L4 strains showed that the four genes also existed in L4 virulent strains except H37Rv; this indicates that they are not relevant to the virulence of H37Rv (Figure S4). We inferred that the four genes might have been lost through IS6110 in the long-term passage of H37Rv.

Five missense SNPs were identified between the H37Rv and H37Ra strains (Table 4). Through comparison with our six clinical virulent strains, two SNPs were eliminated. Of the other three SNPs, two of them (on PhoP and MazG) have been reported and implicated in H37Rv virulence (Ryndak et al., 2008; Lu et al., 2010). We identified a new mutation, C684G (I228M), on the validated virulence factor gene *espK* in H37Ra strains (Table 4). EspK is an ESX-1 secretion-associated protein that is required for virulence, growth in macrophages, and suppression of macrophage inflammatory and immune response (McLaughlin et al., 2007). Thus, this SNP might be involved in the attenuation of virulence in H37Ra.

**Table 4:** **The SNPs between non-virulent H37Ra and virulent H37Rv**.

**Gene^a^**	**Position^b^**	**SNPs^c^**	**Annotation**
Rv0966c	524 (175)	A->G(V->A)	Hypothetical protein
Rv0757(phoP)	656 (219)	C->T(S->L)	Two component system response transcriptional positive regulator PhoP
Rv0658c	224 (75)	A->G(L->P)	Integral membrane protein
Rv1021(mazG)	656 (219)	C->A(A->E)	Nucleoside triphosphate pyrophosphohydrolase
Rv3879c(espK)	684 (228)	G->C(I->M)	ESX-1 secretion-associated protein EspK

a For comparison, the H37Rv genome is used as reference genome;

b The number in the parentheses indicates the mutant position in protein;

c *The letters in the parentheses indicates the amino acid substitutions*.

#### *M. bovis* vs. *M. bovis* BCG

To explore the genetic differences between *M. bovis* and *M. bovis* BCG, we performed a comparative genomic analysis between three *M. bovis* (including two online complete genomes from NCBI GenBank and *M. bovis* 30 genome) and nine *M. bovis* BCG genomes (including eight online complete genomes from NCBI GenBank and *M. bovis* BCG 26 genome; Table S9C). ANI analysis showed a 99.90% identity between *M. bovis* and *M. bovis* BCG strains. Here, we identified 28 *M. bovis* specific genes (Table S10A), six *M. bovis* BCG specific genes (Table S10B), and 308 missense SNPs (Table S11).

**Figure 2:**
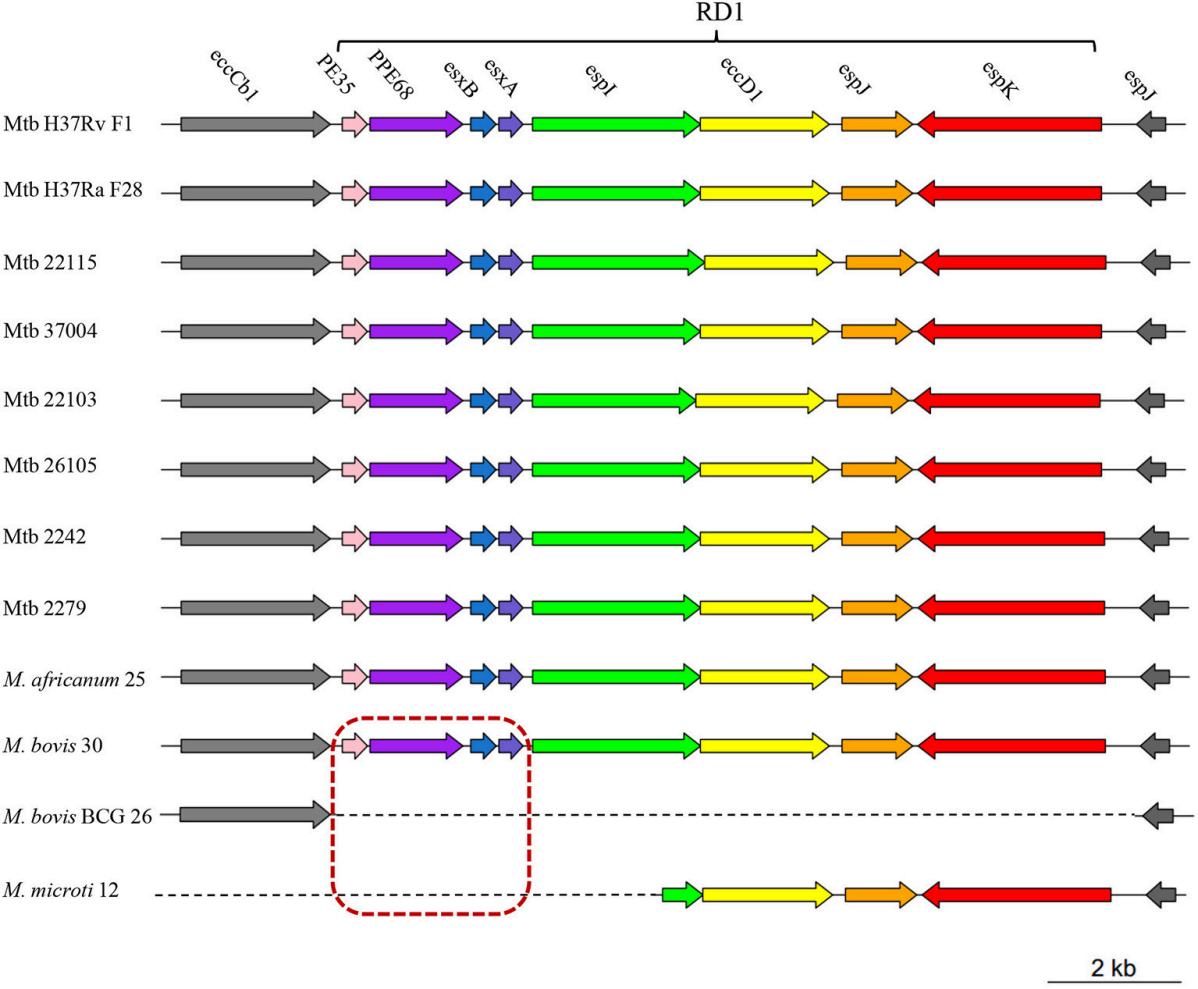
**A schematic diagram showing the RD1 distribution in 12 MTBC strains**. Different colors represent different genes on RD1 (*PE35*, pink; *PPE68*, purple; *esxB*, blue; *esxA*, slate blue; *espI*, green; *eccD1*, yellow; *espJ*, orange; *espK*, red). Genes in the red dashed box indicate the four lost genes in the attenuated *M. bovis* BCG 26 and *M. microti* 12 strains. Genes upstream (*eccCb1*) and downstream (*espJ*) of RD1 are shown in gray.

Eight of 28 *M. bovis* specific genes (i.e., *pe35, ppe68, esxB, esxA, espI, eccD1, espJ*, and *espK*) were clustered together and composed RD1 (Sreejit et al., 2014). Absence of RD1 in *M. bovis* BCG strains was reported to lead to attenuation of virulence (Pym et al., 2002). We further analyzed the distribution of these 8 genes in our 12 MTBC strains and found that only the first four genes (i.e., *pe35, ppe68, esxB*, and *esxA*) were absent in *M. microti* 12 (Figure 2). Since *M. microti* is a non-virulent strain, which seldom causes disease in immunocompetent individuals (Rodriguez-Campos et al., 2014), we deduced that the first four genes of RD1 (rather than RD1) might play a key role in attenuating the virulence of *M. bovis* BCG and *M. microti* strains. In addition, 13 phiRV1 phage proteins were also identified to be absent in *M. bovis* BCG strains. They were clustered together and composed RD3 (Table S10A). It was reported that they could sense oxygen status within the host; thus, their presence could help *M. bovis* adapt to environmental stress (Fan et al., 2016).

Three hundred and eight missense SNPs (on 251 genes) were identified between *M. bovis* and *M. bovis* BCG (Table S11A). Further Clusters of Orthologous Group (COG) functional analysis indicated that 19 genes (19 missense SNPs) displayed a relative enrichment in COG category I (Lipid transport and metabolism; Figure S5). MTBC strains exhibited lipid enrichment on their cell surfaces, which is reported to play a role in pathogenesis (Cole et al., 1998). Importantly, 21 of the nonsynonymous SNPs were located on 21 virulence factors (four validated and 17 putative; Table S11B). These SNPs appear to be associated with the attenuation of virulence in *M. bovis* BCG strains. At last, we need to emphasize that we are particularly careful about the conservation of the standard strains (obtained from ATCC), and they had not been subject to repeated *in vitro* cultivation and belong to the first generations of the original strains. Even so, the animal studies were not carried out due to the limitation of objective condition, all the arguments on the virulence is a speculation that need further validation.

### Antigen epitope variations

An epitope is the part of an antigen that is recognized by the immune system, and plays a core role in immune response (https://en.wikipedia.org/wiki/Epitope). To date, epitope studies are confined to T-cells in *M. tuberculosis* (Comas et al., 2010; Copin et al., 2014; Lindestam Arlehamn et al., 2015). However, comparative analysis of epitopes in different lineages of MTBC strains, including T-cell and B-cell epitopes, is still lacking. In this study, we analyzed the diversity of T-cell and B-cell epitopes in 12 MTBC strains, which serves to enhance our understanding of immune response differences in diverse MTBC strains.

A total of 2,245 MTBC epitopes were downloaded from the IEDB (Vita et al., 2015), including 1,755 T-cell epitopes and 490 B-cell epitopes (Table S12). Pan-genomic analysis indicated that 1,522 epitopes were conserved in all 12 MTBC strains (Figure S2C). Among these, 1,428 epitopes possessed the same copy number.

**Figure 3:**
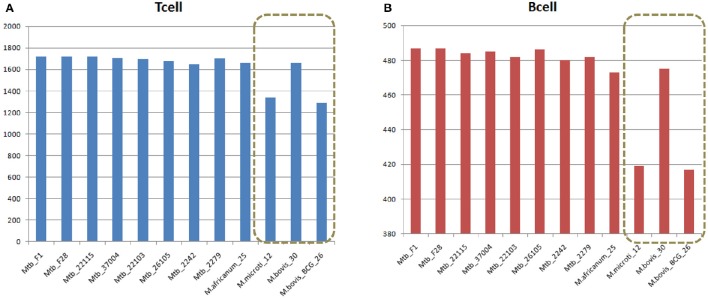
**Comparison of (A)** T cell and **(B)** B cell epitopes in 12 MTBC strains. Duplicate epitopes were removed, and only epitopes with 100% identical matches were considered present in the strain. The x-axis refers to the strain name, and the vertical axis indicates the number of epitopes in the corresponding strain.

Despite sharing the majority of both T-cell and B-cell epitopes, the 12 MTBC strains still had some differences. Two non-virulent animal-origin MTBC strains, *M. bovis BCG* and *M. microti*, had significantly less T-cell and B-cell epitopes, suggesting that these lost epitopes might be related to the non-virulence, since more than 50% of the lost epitopes (164 of 326) were located in the virulence factors (Figure 3, Table S13).

#### Antigen epitope analysis between human- and animal-isolates

**Figure 4:**
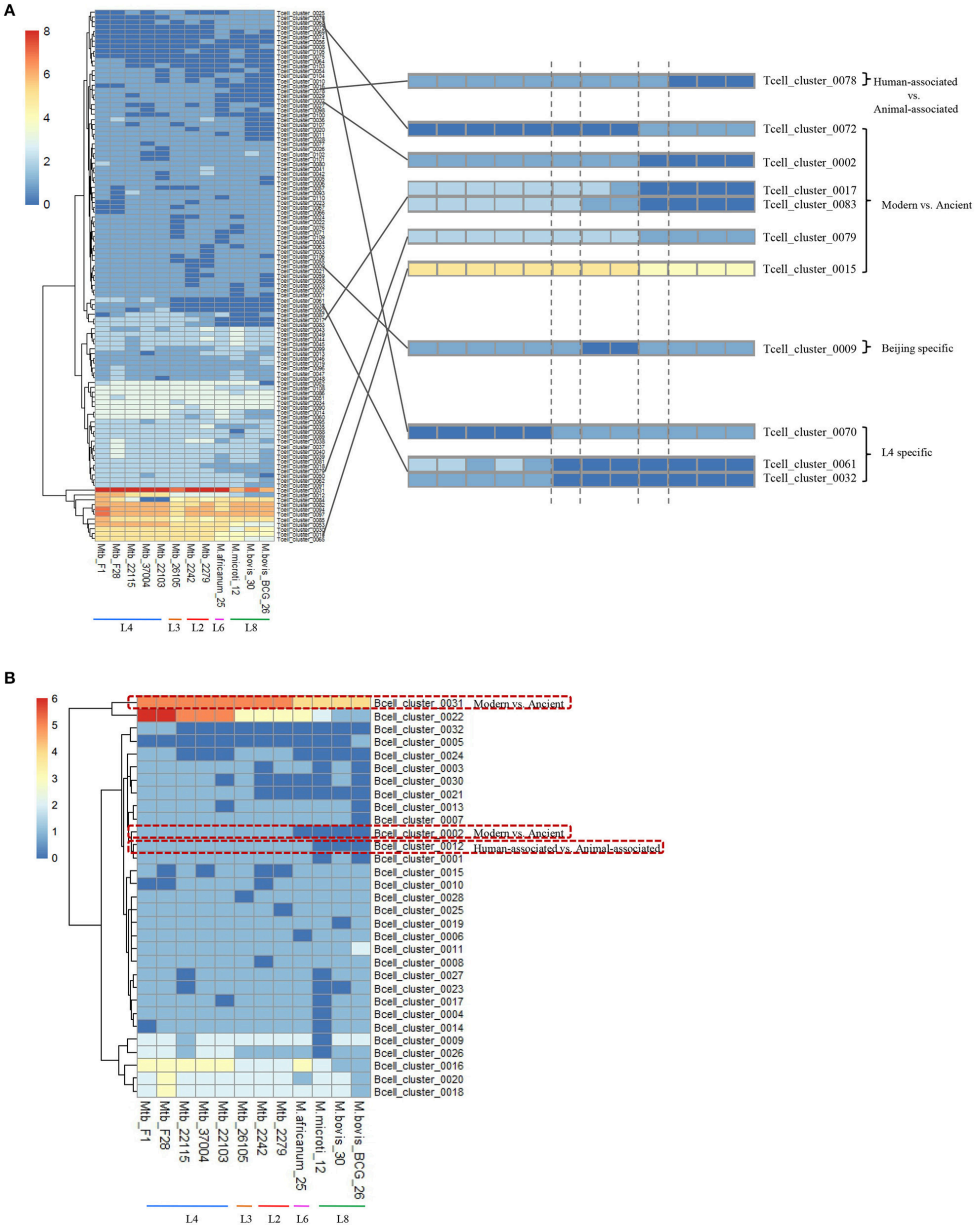
**Distribution of differential T cell (A)** and B cell **(B)** epitope-clusters in 12 MTBC strains. The epitope-clusters present in all 12 MTBC strains with the same copy number were excluded. Each row represents an epitope-cluster, and each column indicates a strain. The color intensity shows the copy number of each epitope-cluster. Some epitope-clusters were enlarged to highlight the differences amongst diverse MTBC lineages on the right section of figure.

The epitopes with the same distribution pattern in the 12 MTBC strains were clustered to further clarify the results. We then analyzed the epitope-clusters for the 12 MTBC strains (Figure 4). For T-cell epitope-clusters, we first investigated the relationship between epitopes and host association. Four epitope-clusters (Tcell_cluster_0002, 0017, 0078, and 0083: 25 epitopes) and one epitope-cluster (Tcell_cluster_0002: two epitopes) were identified to exist only in the human- and animal-associated MTBC strains, respectively (Figure 4A). In addition, the copy number of two epitope-clusters (Tcell_cluster_0015 and 0079: five epitopes) in the human isolates was higher than in the animal isolates. For B cell epitope-clusters, two epitope-clusters (Bcell_cluster_0002 and 0012: eight epitopes) only existed in human isolates, and the copy number of one epitope-cluster (Bcell_cluster_0031: one epitope) in human isolates was higher than in animal-associated ones (Figure 4B).

**Figure 5:**
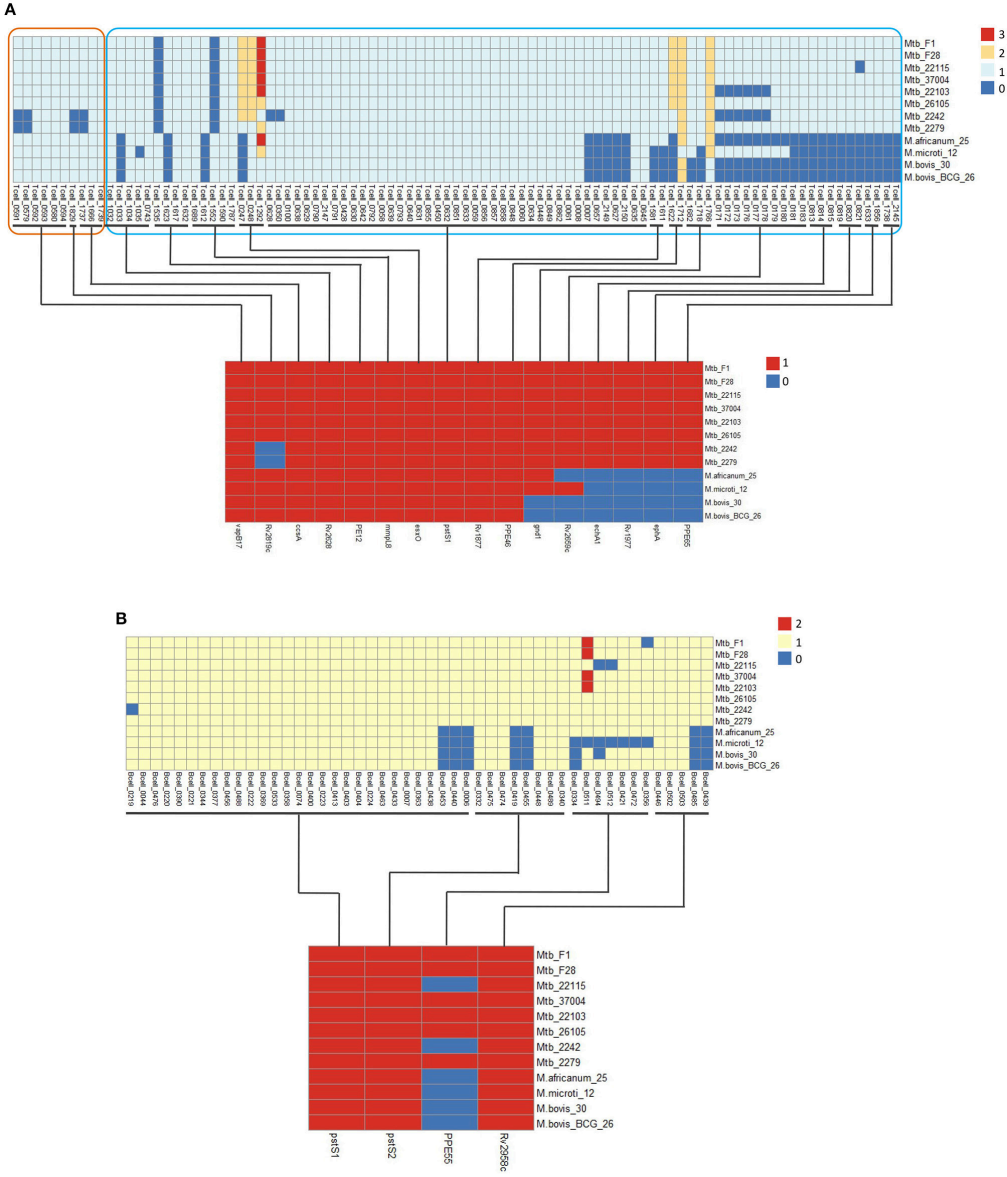
**Distribution of some differential T-cell (A)** and B-cell **(B)** epitopes and the corresponding antigen genes for 12 MTBC strains. The distribution of epitopes and corresponding antigens are shown at the top and bottom sections of the figure. Epitope copy number and antigen is indicated by the intensity of the color.

To further explore the immune-genetic mechanisms, we studied the corresponding antigen genes of the previously described epitopes. The 25 human-associated strain-specific epitopes (belonging to four T-cell epitope-clusters) were located on 13 antigen genes (Figure 5). Among these, the loss of ten epitopes in animal-associated strains resulted from the deletion of the corresponding antigen genes; the loss of the other 15 epitopes was due to SNPs or Insertions or Deletions (Indels) on the relevant antigen genes (Table S14).

The deletion of antigen genes *ephA* (epoxide hydrolase) and *ppe65* (PPE family protein), which are located in RD8, led to the loss of four epitopes (Tcell_1633 and 18566+6; Tcell_1738 and 2145) in animal-associated strains. *EphA* has been reported to regulate integrin-mediated T lymphocyte interactions, and its activation might lead to the down-regulation of T-cell interactions (Sharfe et al., 2008). In addition, three human-associated strain-specific epitopes (Tcell_0813, 0814, and 0815) were located on the antigen gene *echA1* (described in “**Host association analysis**”). *EchA1* was previously reported to be lost in *M. bovis* and *M. bovis BCG* strains (Brosch et al., 2002; Rosenkrands et al., 2008). Our analysis shows that *echA1* is also absent from *M. microti* strains. In addition, our analysis indicates that the animal-associated strains lacked the antigen gene *Rv1977*, which resulted in the absence of epitopes (Tcell_0819-0821).

In addition to the deletion of antigens, there were 15 human-associated strains with specific epitopes attributed to SNPs and Indels in nine T cell antigen genes of animal-associated strains (Table S14). One of these was the antigen gene *PstS-1*, which is a lipoprotein phosphate transport receptor on the cell surface (de Araujo et al., 2014); this possessed five mutant epitopes in animal-associated strains. In our study, the five mutant epitopes were all derived from one SNP (V352A; Figure S6), which might lead to the animal-specific immune reaction.

The 25 human-associated strain-specific epitopes were further tested on ten animal-associated strains (two *M. bovis* and eight *M. bovis BCG* strains) with complete genome sequences from the NCBI (Table S9C). BLAST results confirmed our findings.

#### Antigen epitope analysis of beijing strains

Based on different animal experiments showing the Beijing strains to be more virulent, and to cause more histopathological changes, higher outgrowth, and increased mortality (Ida et al., 2010), the Beijing strains are considered the most pathogenic of the MTBC strains. Epitope-cluster analysis showed that Tcell_cluster_0009 (4 epitopes: Tcell_0579, 0591, 1737, and 1829) was lost only in the Beijing strains (Mtb 2242 and 2279; Figures 4, 5). Sequence alignment results revealed that the nucleotide sequence “AAACATT” from 71 to 77 of the antigen gene *vapB17* mutated into “AC” in these two strains; this resulted in the absence of epitope Tcell_0579 and 0591. The “I245M” mutation on antigen CcsA led to the loss of epitope Tcell_1737. Finally the deletion of the antigen gene *Rv2819c* (a CRISPR type III-associated RAMP protein Csm5) led to the loss of epitope Tcell_1829 in the Beijing lineage strains. Further, verification was carried out on ten other Beijing lineage strains with complete genome sequences obtained from NCBI (Table S9A). The results confirmed the finding that these four epitopes were lost in all Beijing lineage strains. The absence of the four epitopes in all of these Beijing lineage strains might enable them to better evade the human immune system and promote growth in the hostile environment of the host cell. This could be a reason for their more virulent phenotype.

#### Antigen epitope analysis between *M. bovis* and *M. bovis* BCG strains

To investigate the relationship between epitope variation and virulence, we compared the difference in epitopes of three virulent *M. bovis* strains and nine non-virulent *M. bovis* BCG strains (Figure S7). We determined that Tcell_cluster_A02 (149 epitopes) and Bcell_cluster_A01 (19 epitopes) were lost in all non-virulent BCG strains; meanwhile, Tcell_cluster_A06 (six epitopes) and Bcell_cluster_A04 (one epitope) were absent from all *M. bovis* strains. Tcell_cluster_A23 (five epitopes) had two copies in *M. bovis*, but only one copy in *M. bovis* BCG. We further examined the 13 corresponding antigen genes carrying the sequences of the above 180 epitopes (Figure S8 and Table S15). Among these, the loss of 146 epitopes (including 130 T-cell epitopes and 16 B-cell epitopes) in BCG strains resulted from the deletion of the corresponding three antigen genes (*esxA, esxB*, and *ppe68*), which were located in RD1 and are reported to be associated with the attenuation of virulence in *M. bovis* BCG (Lewis et al., 2003).

## Conclusion

Tuberculosis now exceeds HIV as the top infectious disease killer, and is caused by the *Mycobacterium tuberculosis* complex (MTBC). MTBC strains possess highly similar genomes (99%) but large variations in phenotype, including host-association, virulence, and immunoreactivity. This might be the result of long-term co-evolution with diverse populations of humans and animals. Long-term geographical isolation gave rise to the accumulation of “genotype isolation,” thus resulting in different phenotypes. To analyze the relationship between genotype and phenotype, we selected 12 MTBC strains representing different lineages, re-sequenced their genomes to correct homo-polymer errors, and performed a comparative genomic analysis of the strains.

Host-association analysis indicated that eight *mce3* family genes, two enoyl-CoA hydratases, and five PE/PPE family genes were present in human-associated strains but not in animal-associated strains.

Fifteen SNPs were found on virulence factors (including five SNPs in three ESX secretion-proteins) only in the Beijing strains. Comparisons between the virulent H37Rv strain and the non-virulent H37Ra strain revealed that three SNPs, S219L (PhoP), A219E (MazG), and a newly identified I228M (EspK), might lead to the attenuation of virulence in H37Ra. On the other hand, a comparison of the animal-associated MTBC strains showed that the deletion of the first four genes (i.e., *pe35, ppe68, esxB*, and *esxA*), rather than all eight RD1 genes, might play a central role in the virulence of animal-associated MTBC strains.

Finally, by comparing the epitopes of MTBC strains, we found that four epitopes were lost only in the Beijing strains. In addition, 32 T-cell epitopes and eight B-cell epitopes showed different distribution patterns in human- and animal-associated strains.

Overall, our research reveals the genetic variations leading to the differences in MTBC genotypes, and enhances our understanding of the relationship between highly conserved genome sequences/genotypes (similarity >99%) and highly different phenotypes in MTBC strains.

## Accession numbers

The SRA accession number for the sequencing data reported in this paper is SRP064893. The GAS (Genome Sequence Archive in BIG Data Center) accession number is PRJCA000307.

## Author contributions

FC, XJ, and HH designed the project. XJ, MD, and FC wrote the paper. XJ, LY, SC, LL, DC, JF, TY, JZ, XZ, YS, GW, and YS performed the experiments.

### Conflict of interest statement

The authors declare that the research was conducted in the absence of any commercial or financial relationships that could be construed as a potential conflict of interest.
