# Supplementary material for: The Bioinformatics Analysis of Comparative Genomics of Mycobacterium tuberculosis Complex (MTBC) Provides Insight into Dissimilarities between Intraspecific Groups Differing in Host Association, Virulence, and Epitope Diversity
Source: Front Cell Infect Microbiol. 2017 Mar 21;7:88. doi: 10.3389/fcimb.2017.00088 (PMC5360109; doi:10.3389/fcimb.2017.00088)
Supplement: Supplementary file 1 [file DataSheet1.zip › Supplementary.doc]

Supplementary Material:

**The bioinformatics analysis of comparative genomics of *Mycobacterium tuberculosis* complex (MTBC) provides insight into dissimilarities between intraspecific groups differing in host association, virulence and epitope diversity**

Xinmiao Jia^1, 2#^, Li Yang^1, 2#^, Mengxing Dong^1, 2#^, Suting Chen^3#^, Lingna Lv^3#^, Dandan Cao^1^, Jing Fu^1, 2^, Tingting Yang^1, 2^, Ju Zhang^1^, Xiangli Zhang^1, 2^, Yuanyuan Shang^3^, Guirong Wang^3^, Yongjie Sheng^4^, Hairong Huang^3^*, Fei Chen^1,2,5,6^*

^#^These authors contributed equally to this work.

*To whom correspondence should be addressed. Tel: +86 10 8409 7460; Fax: +86 10 8409 7845; E-mail: chenfei@big.ac.cn

*Correspondence may also be addressed to Prof. Hairong Huang. Tel: 86-10-89509359; Fax: 86-10-89509359; E-mail: huanghairong@tb123.org

**1 Supplementary Data**

Perl script used to parse MAUVE generated SNPs (This is an example to retrieve SNPs associated to different hosts).

#!/usr/bin/perl -w

open IN,"<12MTBC_corrected_mauve_add962.snps"or die "can not write file:$!";

open OUT,">SNPs_Diff_hosts.snps"or die "can not write file:$!";

print OUT "Ref_pos\t"."REF\t"."ALT\n";

while(<IN>){

chomp($_);

if (/^SNP/) {}

else{

my @lines=split /\t+/,$_;

my $snps_pattern=$lines[0];

my $pos_962=$lines[1];

my $Ref=substr($snps_pattern,0,1);

my $F1=substr($snps_pattern,1,1);

my $F28=substr($snps_pattern,2,1);

my $t22103=substr($snps_pattern,3,1);

my $t22115=substr($snps_pattern,4,1);

my $t37004=substr($snps_pattern,5,1);

my $t26105=substr($snps_pattern,6,1);

my $t2242=substr($snps_pattern,7,1);

my $t2279=substr($snps_pattern,8,1);

my $t25=substr($snps_pattern,9,1);

my $t12=substr($snps_pattern,10,1);

my $t26=substr($snps_pattern,11,1);

my $t30=substr($snps_pattern,12,1);

if ($F28 eq $F1 && $t22103 eq $F1 && $t22115 eq $F1 && $t37004 eq $F1 && $t26105 eq $F1 && $t2242 eq $F1 && $t2279 eq $F1 && $t26 eq $t12 && $t30 eq $t12 && $F1 ne $t12) {

print OUT "$pos_962\t"."$Ref\t"."$t30\n";

}

}

}

close IN;

close OUT;

#!/usr/bin/perl -w

#SNPs details

my $version=1.00;

use strict;

use Getopt::Long;

my %opts;

GetOptions(\%opts,"v=s","a=s","g=s","o=s","h");

if (!(defined $opts{v} and defined $opts{a} and defined $opts{g} and defined $opts{o}) || defined $opts{h}) {

&usage;

}

my $filev=$opts{v};

my $filea=$opts{a};

my $fileg=$opts{g};

my $fileo=$opts{o};

open SNV,"<$filev";

open ORF,"<$filea";

open SEQ,"<$fileg";

open OUT,">$fileo";

print OUT "PosOnGenome\t"."SNPs Type\t"."GR/IGR\t"."GeneStart\t"."GeneEnd\t"."Strand\t"."GeneName\t"."Synonym\t"."Product\t"."Synonymous/Nonsynonymous\t"."PosOnGene\t"."PosOnPep\t"."AA mutation\n";

my %snv=();

while(<SNV>){

chomp();

if (/^[0-9]/) {

my @lines=split /\t+/,$_;

if (length($lines[1]) == 1 && length($lines[2]) == 1 ) {

my $key="$lines[0]";

my $type="$lines[1]".'->'."$lines[2]";

$snv{$key}=$type;

}

}

}

my %orf=();

my $num=1;

while (<ORF>) {

chomp();

if (/^[0-9]/) {

my @anno=split /\t+/,$_;

my $strand=$anno[1];

my $name=$anno[4];

my $product=$anno[8];

my $loc=$anno[0];

my @pos=split /\.+/,$loc;

my $start=$pos[0];

my $end=$pos[1];

$orf{$num}="$start\t"."$end\t"."$strand\t"."$name\t"."$anno[5]\t"."$product";

$num+=1;

}

}

my %coding=();

my %noncoding=();

my %RNAs=();

foreach my $str (keys %snv) {

my $location="";

my $switch=0;

for (my $i=1; $i<=3976; $i++) {

my @array=split /\t+/,$orf{$i};

if ($str <= $array[1] && $str >= $array[0]) {

if ($array[2] eq '+') {

$location="$str\t"."$array[0]".'-'."$array[1]";

}

else{

$location="$str\t"."c"."$array[1]".'-'."$array[0]";

}

if ($i <= 3906 && $i >= 1) {

$coding{$location}="$str\t"."$snv{$str}\t"."GeneCodingRegion\t"."$orf{$i}";

#print OUT "$str\t"."$snv{$str}\t"."GeneCodingRegion\t"."$orf{$i}\n";

#print $location;

}

else{

$RNAs{$str}="$str\t"."$snv{$str}\t"."RNAs\t"."$orf{$i}";

#print OUT "$str\t"."$snv{$str}\t"."RNAs\t"."$orf{$i}\n";

}

$i=8000;

$switch=1;

}

}

if ($switch == 0) {

$noncoding{$str}="$str\t"."$snv{$str}\t"."InterGenicRegion";

#print OUT "$str\t"."$snv{$str}\t"."NonCodingRegion\n";

}

}

my %seq=();

my $key_pos="";

while (<SEQ>) {

chomp();

if (/^>/) {

my $colon=':';

my $name=' Mycobacterium tuberculosis';

my $len=index($_,$name)-index($_,$colon)-1;

my $start=index($_,$colon)+1;

$key_pos=substr($_,$start,$len);

$seq{$key_pos}="";

#print "$key_pos\n";

}

else{

$seq{$key_pos}="$seq{$key_pos}"."$_";

}

}

my %CodonAA=();

$CodonAA{"TTT"}=$CodonAA{"TTC"}="F";

$CodonAA{"TTA"}=$CodonAA{"TTG"}=$CodonAA{"CTT"}=$CodonAA{"CTC"}=$CodonAA{"CTA"}=$CodonAA{"CTG"}="L";

$CodonAA{"ATT"}=$CodonAA{"ATC"}=$CodonAA{"ATA"}="I";

$CodonAA{"ATG"}="M";

$CodonAA{"GTT"}=$CodonAA{"GTC"}=$CodonAA{"GTA"}=$CodonAA{"GTG"}="V";

$CodonAA{"TCT"}=$CodonAA{"TCC"}=$CodonAA{"TCA"}=$CodonAA{"TCG"}=$CodonAA{"AGT"}=$CodonAA{"AGC"}="S";

$CodonAA{"CCT"}=$CodonAA{"CCC"}=$CodonAA{"CCA"}=$CodonAA{"CCG"}="P";

$CodonAA{"ACT"}=$CodonAA{"ACC"}=$CodonAA{"ACA"}=$CodonAA{"ACG"}="T";

$CodonAA{"GCT"}=$CodonAA{"GCC"}=$CodonAA{"GCA"}=$CodonAA{"GCG"}="A";

$CodonAA{"TAT"}=$CodonAA{"TAC"}="Y";

$CodonAA{"CAT"}=$CodonAA{"CAC"}="H";

$CodonAA{"CAA"}=$CodonAA{"CAG"}="Q";

$CodonAA{"AAT"}=$CodonAA{"AAC"}="N";

$CodonAA{"AAA"}=$CodonAA{"AAG"}="K";

$CodonAA{"GAT"}=$CodonAA{"GAC"}="D";

$CodonAA{"GAA"}=$CodonAA{"GAG"}="E";

$CodonAA{"TGT"}=$CodonAA{"TGC"}="C";

$CodonAA{"TGG"}="W";

$CodonAA{"CGT"}=$CodonAA{"CGC"}=$CodonAA{"CGA"}=$CodonAA{"CGG"}=$CodonAA{"AGA"}=$CodonAA{"AGG"}="R";

$CodonAA{"GGT"}=$CodonAA{"GGC"}=$CodonAA{"GGA"}=$CodonAA{"GGG"}="G";

$CodonAA{"TAA"}=$CodonAA{"TAG"}=$CodonAA{"TGA"}="end";

my %minus=();

$minus{"A"}="T";

$minus{"T"}="A";

$minus{"G"}="C";

$minus{"C"}="G";

my $LocOnGene=0;

my $codon="";

my $editing_codon="";

my $mutation_type="";

my $remainder=0;

my $LocOnPep=0;

foreach my $char (keys %coding) {

my @info=split /\t+/,$coding{$char};

my $snv_site=$info[0];

my $gene_s=$info[3];

my $gene_e=$info[4];

my $strands=$info[5];

my @locs=split /\t+/,$char;

my $gene_seq=$seq{$locs[1]};

my $editing_base=substr($info[1],3,1);

if ($strands eq '+') {

$LocOnGene=$snv_site-$gene_s+1;

$remainder=$LocOnGene%3;

if ($remainder == 1) {

$codon=substr($gene_seq,$LocOnGene-1,3);

$editing_codon="$editing_base".substr($codon,1,2);

#print "$editing_codon\t"."$codon\n";

$LocOnPep=($LocOnGene-1)/3 + 1;

}

elsif($remainder == 2){

$codon=substr($gene_seq,$LocOnGene-2,3);

$editing_codon=substr($codon,0,1)."$editing_base".substr($codon,2,1);

#print "$codon\t"."$editing_codon\n";

#print "$snv_site\t"."$gene_s\t"."$gene_e\t"."$LocOnGene\n";

$LocOnPep=($LocOnGene-2)/3 + 1;

}

else{

$codon=substr($gene_seq,$LocOnGene-3,3);

$editing_codon=substr($codon,0,2)."$editing_base";

$LocOnPep=$LocOnGene/3;

}

}

else{

$LocOnGene=$gene_e-$snv_site+1;

$remainder=$LocOnGene%3;

if ($remainder == 1) {

$codon=substr($gene_seq,$LocOnGene-1,3);

$editing_codon="$minus{$editing_base}".substr($codon,1,2);

#print "$editing_codon\t"."$codon\n";

$LocOnPep=($LocOnGene-1)/3 + 1;

}

elsif($remainder == 2){

$codon=substr($gene_seq,$LocOnGene-2,3);

$editing_codon=substr($codon,0,1)."$minus{$editing_base}".substr($codon,2,1);

#print "$codon\t"."$editing_codon\n";

#print "$snv_site\t"."$gene_s\t"."$gene_e\t"."$LocOnGene\n";

$LocOnPep=($LocOnGene-2)/3 + 1;

}

else{

$codon=substr($gene_seq,$LocOnGene-3,3);

$editing_codon=substr($codon,0,2)."$minus{$editing_base}";

$LocOnPep=$LocOnGene/3;

}

}

if ($CodonAA{$codon} eq $CodonAA{$editing_codon}) {

$mutation_type="synonymous\t"."$LocOnGene";

#print "$codon\t"."$editing_codon\n";

}

else{

$mutation_type="nonsynonymous\t"."$LocOnGene\t"."$LocOnPep\t"."$CodonAA{$codon}"."->"."$CodonAA{$editing_codon}";

}

if ($strands eq '+') {

print OUT "$coding{$char}\t"."$mutation_type\n";

}

else{

my $edited=$minus{substr($info[1],3,1)};

my $original=$minus{substr($info[1],0,1)};

print OUT "$info[0]\t"."$original".'->'."$edited\t"."$info[2]\t"."$info[3]\t"."$info[4]\t"."$info[5]\t"."$info[6]\t"."$info[7]\t"."$info[8]\t"."$mutation_type\n";

}

}

foreach my $rna (keys %RNAs) {

my @info=split /\t+/,$RNAs{$rna};

my $strands=$info[8];

if ($strands eq '+') {

print OUT "$RNAs{$rna}\n";

}

else{

my $edited=$minus{substr($info[1],3,1)};

my $original=$minus{substr($info[1],0,1)};

print OUT "$info[0]\t"."$original".'->'."$edited\t"."$info[2]\t"."$info[3]\t"."$info[4]\t"."$info[5]\t"."$info[6]\t"."$info[7]\t"."$info[8]\n";

}

}

foreach my $non (keys %noncoding) {

print OUT "$noncoding{$non}\n";

}

close SNV;

close ORF;

close SEQ;

close OUT;

sub usage{

print <<"USAGE";

Version $version

Usage:

$0 -v -a -g -o

options:

-v SNVs file(vcf file)

-a annotation file(ptt rnt file)

-g gene sequence file(ffn file)

-o output file

-h help

USAGE

exit(1);

}

**2 Supplementary Figures and tables**

**2.1 Figures**

**Figure S1.** Phylogenetic analysis of *Mycobacterium tuberculosis* complex (MTBC) strains. Blue indicates that strains belong to lineage 4 (L4, Euro-American lineage). Pink indicates that strains belong to L3 (East African-Indian lineage). Red indicates that strains belong to L2 (East Asian lineage). Orange indicates that strains belong to L1 (Indo-Oceanic lineage). Purple indicates that strains belong to L5 and L6 (West African 1 and West African 2 lineage). Dark blue indicates that strains belong to L7 (Ethiopia lineage). Green indicates that strains belong to animal-adapted lineage (L8). The strains in red boxes represent the 12 MTBC genomes we finished here (Zhu et al., 2016).

**Figure S2.** (A) Flower plots showing the core gene number (in the center), dispensable gene number (in the annulus) and strain-specific gene number (in the petals) in 12 MTBC strains. The numbers under the strain name represent the total number of coding proteins. (B) Flower plots showing the core virulence factor (VF) number (in the center), dispensable VF number (in the annulus) and strain-specific VF number (in the petals) in 12 MTBC strains. The numbers under the strain name represent the total number of VFs. (C) Flower plots showing the core epitope number (in the center), dispensable epitope number (in the annulus) and strain-specific epitope number (in the petals) in 12 MTBC strains. The numbers under the strain name represent the total number of epitopes. Different colors indicate different lineage strains: L4 strains, green; L3 strain, yellow; L2 strains, red; L6 strain, blue; L8 strains, plum.

**Figure S3.** Schematic diagram showing the 14 lost genes on RD7 in animal isolates. Eight *mce3* are marked with different colors (*mce3A*, red; *mce3B*, orange; *mce3C*, yellow; *mce3D*, greenyellow; *plrM*, cyan; *mce3F*, purple; *Rv1972*, magenta; *Rv1973*, hotpink). The two lost genes upstream (*yrbEA* and y*rbEB*) and four lost genes downstream (*Rv1974*, *Rv1975*, *Rv1976c* and *Rv1977*) of *mce3* are marked as green with different color gradations. In addition, the genes upstream (*Rv1963c*) and downstream (*Rv1978*) of RD7 are shown as grey.

**Figure S4.** Schematic diagram showing that H37Ra has four extra genes (the *plcD*-*cut1* region (RvD2)) in comparison with H37Rv. Different colors represent different genes (*plcD*, dark grey; IS6110, neutral grey; *MRA_1768A*, red; *MRA_1768B*, yellow; *MRA_1768C*, green; *mmpL14*, blue;*cut1*, light grey). Three L4 strains (Mtb 22115, 37004 and 22103) are listed below as references.

**Figure S5.** Functional classes of genes containing the SNPs between *M. bovis* and *M. bovis* BCG across COG categories. The y-axis shows the number of genes in each functional class. The x-axis shows the functional classes.

**Figure S6.** Missense mutation (V->A) on antigen PstS1 results in the losses of five epitopes in animal isolates. Five epitopes are in different color-coded boxes (Tcell_0007, red; Tcell_0657, orange; Tcell_2149, green; Tcell_0627, purple; Tcell_2150, blue).

**Figure S7.** Distribution of epitope-clusters of T cell (A) and B cell (B) in *M. bovis* and *M. bovis* BCG. Each row represents an epitope-cluster, and each column indicates a strain. The color intensity shows the copy number of each epitope-cluster.

**Figure S8.** Distribution of some differential epitopes and the relative antigen genes of T cell (A) and B cell (B) in *M. bovis* and *M. bovis* BCG. The distribution of epitopes and antigens are shown at the top and bottom sections of the figure. The copy number of epitope and antigen is displayed by the color intensity.

**2.2 Tables**

**Table S1.** The information of 12 MTBC strains used in the study.

A. Reference strains

| **Strain No.** | **Species** | **ATCC No.** | **Lineage** |
| --- | --- | --- | --- |
| **F1** | *M. tuberculosis* H37Rv | 27294 | L4 |
| **F28** | *M. tuberculosis* H37Ra | 25177 | L4 |
| **25** | *M. africanum* | 35711 | L6 |
| **12** | *M. microti* | 19422 | L8 |
| **30** | *M.bovis* | 19210 | L8 |
| **26** | *M.bovis* BCG | 35735 | L8 |

B. Clinical strains

| **Strain No.** | **Species** | **VNTR15China** | **Pks15/1** | **Lineage** | **Source** | **Date** | **Patient Sex** | **Patient Age** | **Disease Diagnosis** | **Sample Type** |
| --- | --- | --- | --- | --- | --- | --- | --- | --- | --- | --- |
| 2242 | *M. tuberculosis* | 442333504465382 |  | L2 | Beijing Chest Hospital | 2006 | Male | 37 | Tuberculosis | Sputum |
| 2279 | *M. tuberculosis* | 442333474455392 |  | L2 | Beijing Chest Hospital | 2006 | Female | 42 | Tuberculosis | Sputum |
| 22115 | *M. tuberculosis* | 242323343253372 | Δ7 bp/Δ7 bp | L4 | Baseline survey^a^ | 2007.4.30 | Male | 39 | Tuberculosis | Sputum |
| 22103 | *M. tuberculosis* | 443251313413384 | Δ7 bp/Δ7 bp | L4 | Baseline survey | 2007.4.8 | Male | 66 | Tuberculosis | Sputum |
| 37004 | *M. tuberculosis* | 545322354253383 | Δ7 bp/Δ7 bp | L4 | Beijing Chest Hospital | 2010.4.6 | Male | 41 | Knee tuberculosis | Pus |
| 26105 | *M. tuberculosis* | 442374424474492 |  | L3 | Baseline survey | 2007.4.1 | Male | 67 | Tuberculosis | Sputum |

^a^National Survey of Drug-Resistant Tuberculosis in China (Zhao et al., 2012).

**Table S2. Oligonucleotide primers used in the study.**

| **Primer name** | **Sequence (5’→3’)** |
| --- | --- |
| pks15/1-F | ATCTCGCCGAAATCACCCACG |
| pks15/1-R | CGTACCAGCCCCCGCAGAG |
| Type IIG-NsiI-F | CCAATGCATTTAAGGTTAATCATATGGGGTCGGTGCACGAC |
| Type IIG-BglII-R | GAAGATCTAGCGCTGCGTGTCACGTTGAATCCGAACCCG |
| RC-Type IIG-BglII-R | CGGGTTCGGATTCAACGTGACACGCAGCGCTAGATCTTC |
| S2-MS1-sbfI-F | CGCCTGCAGGTTAAGGTTAATCATATGAGCCGCGTCGAGAAAG |
| S2-MS1-BamHI-R | CGGGATCCCTGCGTGGTATACTGATCACTAACGGGGATCGTTGCCC |
| S2MS1-AscI-F | GGCGCGCCCCTTCGTGTTGTCGAGGAG |
| S2MS1-AscI-R | GGCGCGCCTGCGCTGGCTCTTGATATT |
| MS1-AscI-F | GGCGCGCCTTAAGGTTAATCATATGCCGCCCAGGAAGAAGCA |
| MS1-AscI-R | GGCGCGCCATGATTAACCTTAACCTGCAGG |
| S2M-KpnI-F | GGGGTACCTGATCAGTATACCACGCAGGG |
| S2M-KpnI-F | GGGGTACCTCATCTAAGCCGCCCCAGTTG |
| PRRS-F1 | GCCGCAGTGTTATCACTCATG |
| PRRS-F2 | GGCTTTACACTTTATGCTTCCG |
| PRRS-R | GGGTTTTCCCAGTCACGACG |
| F: forward primer; R: reverse primer. |  |
| Restriction sites used for cloning are underlined, and sequences used for diagnostic purposes are double underlined. | |

**Table S3.** 257 MTBC virulence factors collected from VFDB.

A. Experimentally validated virulence factors

| ahpC  (Rv2428) ^a^ | mbtB (Rv2383c) | proC (Rv0500) | hspR(Rv0353) | esxM (Rv1792) | mmpL7 (Rv2942) | eccE5 (Rv1797) | fadD26 (Rv2930) | mycP1 (Rv3883c) | espD (Rv3614c) |
| --- | --- | --- | --- | --- | --- | --- | --- | --- | --- |
| devR/dosR (Rv3133c) | mbtC (Rv2382c) | prrA (Rv0903c) | fbpD(Rv3803c) | esxN (Rv1793) | mprA (Rv0981) | eccA5 (Rv1798) | fadD33 (Rv1345) | narG (Rv1161) | eccA1 (Rv3868) |
| devS (Rv3132c) | mbtD (Rv2381c) | purC (Rv0780) | esxB (Rv3874) | mmaA4 (Rv0642c) | eccD5 (Rv1795) | fbpA (Rv3804c) | narH (Rv1162) | eccB1 (Rv3869) | espK (Rv3879c) |
| drrC (Rv2938) | mbtE (Rv2380c) | relA (Rv2583c) | fadD28 (Rv2941) | mycP5 (Rv1796) | espC (Rv3615c) | fbpB (Rv1886c) | narI (Rv1164) | eccCa1 (Rv3870) | panC (Rv3602c) |
| erp (Rv3810) | mbtF (Rv2379c) | eccB5 (Rv1782) | leuD (Rv2987c) | plcA (Rv2351c) | sigH (Rv3223c) | hspX (Rv2031c) | panD (Rv3601c) | eccE1 (Rv3882c) | hbhA (Rv0475) |
| espA (Rv3616c) | mbtG (Rv2378c) | eccCa5 (Rv1783) | lipF (Rv3487c) | plcB (Rv2350c) | sodA (Rv3846) | icl (Rv0467) | pcaA (Rv0470c) | sigA/rpoV (Rv2703) | eccCb1 (Rv3871) |
| espB (Rv3881c) | mbtH (Rv2377c) | eccCb5 (Rv1784) | lpqH (Rv3763) | plcC (Rv2349c) | sodC (Rv0432) | ideR (Rv2711) | PE35 (Rv3872) | sigE (Rv1221) | narJ (Rv1163) |
| esxA (Rv3875) | mgtC (Rv1811) | Rv1794 | mas (Rv2940c) | plcD (Rv1755c) | trpD (Rv2192c) | katG (Rv1908c) | phoP (Rv0757) | sigF (Rv3286c) | fbpC (Rv0129c) |
| glnA1 (Rv2220) | ompA (Rv0899) | eccD1 (Rv3877) | mbtA (Rv2384) | PPE41 (Rv2430c) | whiB3 (Rv3416) |  |  |  |  |

^a^The letters in the parentheses indicates the corresponding gene ID in H37Rv (NC_000962).

B. Homologous virulence factors

| mce1E (Rv0173) | espF (Rv3865) | icl2 (Rv1916) | mce3D (Rv1969) | papA2 (Rv3820c) | regX3 (Rv0491) | sugB (Rv1237) | PPE68 (Rv3873) | sap (Rv3821) | ppsD (Rv2934) |
| --- | --- | --- | --- | --- | --- | --- | --- | --- | --- |
| mps1 (Rv0101) | espG1 (Rv3866) | irtA (Rv1348) | mce3E (Rv1970) | papA3 (Rv1182) | rmlA (Rv0334) | sugC (Rv1238) | PPE69 (Rv3892c) | sapM (Rv3310) | ppsE (Rv2935) |
| adhD (Rv3086) | espG2 (Rv3889c) | irtB (Rv1349) | mce3F (Rv1971) | papA5 (Rv2939) | Rv0203 | tesA (Rv2928) | ppsA (Rv2931) | secA2 (Rv1821) | sigL (Rv0735) |
| caeA (Rv2224c) | espG3 (Rv0289) | kasB (Rv2246) | mce4A (Rv3499c) | pe (Rv1184c) | Rv1344 | tgs4 (Rv3088) | ppsB (Rv2932) | senX3 (Rv0490) | sigM (Rv3911) |
| cccD4 (Rv3448) | espH (Rv3867) | kefB (Rv3236c) | mce4B (Rv3498c) | PE_PGRS30 (Rv1651c) | Rv1786 | zmp1 (Rv0198c) | ppsC (Rv2933) | sigD (Rv3414c) | stf0 (Rv0295c) |
| chp (Rv3087) | espI (Rv3876) | lipR (Rv3084) | mce4C (Rv3497c) | PE18 (Rv1788) | Rv2949c | eccE3 (Rv0292) | gtf1 (Rv1526c) | mce3A (Rv1966) | nuoG (Rv3151) |
| chp1 (Rv3822) | espJ (Rv3878) | lppx (Rv2945c) | mce4D (Rv3496c) | PE19 (Rv1791) | Rv2951c | eis (Rv2416c) | gtf2 (Rv1524) | mce3B (Rv1967) | pafA (Rv2097c) |
| cmaA2 (Rv0503c) | espL (Rv3880c) | lpqY (Rv1235) | mce4E (Rv3495c) | PE36 (Rv3893c) | Rv2952 | espE (Rv3864) | icl2 (Rv1916) | mce3C (Rv1968) | papA1 (Rv3824c) |
| ctpC (Rv3270) | espR (Rv3849) | lprG (Rv1411c) | mce4F (Rv3494c) | PE5 (Rv0285) | Rv2953 | eccC2 (Rv3894c) | fadD29 (Rv2950c) | mce2A (Rv0589) | mycP3 (Rv0291) |
| ctpV (Rv0969) | esxC (Rv3890c) | lysA (Rv1293) | mmpL10 (Rv1183) | phoR (Rv0758) | Rv2954c | eccC3 (Rv0284) | fadE14 (Rv1346) | mce2B (Rv0590) | mycP4 (Rv3449) |
| cyp125 (Rv3545c) | esxD (Rv3891c) | mbtI (Rv2386c) | mmpL11 (Rv0202c) | pknG (Rv0410c) | Rv2955c | eccC4 (Rv3447c) | fadE28 (Rv3544c) | mce2C (Rv0591) | mymA (Rv3083) |
| cyp143 (Rv1785c) | esxG (Rv0287) | mbtJ (Rv2385) | mmpL3 (Rv0206c) | pks1 (Rv2946c) | Rv2956 | eccD2 (Rv3887c) | fadE29 (Rv3543c) | mce2D (Rv0592) | narK2 (Rv1737c) |
| ddrA (Rv2936) | esxH (Rv0288) | mbtK (Rv1347c) | mmpL8 (Rv3823c) | pks15 (Rv2947c) | Rv2957 | eccD3 (Rv0290) | fadE5 (Rv0244c) | mce2E (Rv0593) | narX (Rv1736c) |
| ddrB (Rv2937) | esxT (Rv3444c) | mce1A (Rv0169) | mmpS4 (Rv0451c) | pks15/1 | Rv2958c | eccE2 (Rv3885c) | fmt | mce2F (Rv0594) | ndk (Rv2445c) |
| eccA2 (Rv3884c) | esxU (Rv3445c) | mce1B (Rv0170) | mosR (Rv0348) | pks2 (Rv3825c) | Rv2959c | prrB (Rv0902c) | ptpA (Rv2234) | sugA (Rv1236) | fadD13 (Rv3089) |
| eccA3 (Rv0282) | fad23 (Rv1185c) | mce1C (Rv0171) | mpa (Rv2115c) | PPE25 (Rv1787) | Rv2962c | eccB4 (Rv3450c) | fadD22 (Rv2948c) | mce1F (Rv0174) | mycP2 (Rv3886c) |
| eccB2 (Rv3895c) | fad23 (Rv3826) | mce1D (Rv0172) | mprB (Rv0982) | PPE26 (Rv1789) | Rv3446c | PPE4 (Rv0286) | sadH (Rv3085) | PPE27 (Rv1790) | Rv3888c |
| eccB3 (Rv0283) |  |  |  |  |  |  |  |  |  |

**Table S4.** 167 PE/PPE genes analyzed in this paper.

| PE1  (Rv0151c) ^a^ | PE20  (Rv1806) | PE_PGRS4  (Rv0279c) | PE_PGRS22  (Rv1091) | PE_PGRS39  (Rv2340c) | PE_PGRS57  (Rv3514) | PPE13  (Rv0878c) | PPE30  (Rv1802) | PPE51  (Rv3136) | PPE68  (Rv3873) |
| --- | --- | --- | --- | --- | --- | --- | --- | --- | --- |
| PE2  (Rv0152c) | PE22  (Rv2107) | PE_PGRS5  (Rv0297) | PE_PGRS23  (Rv1243c) | PE_PGRS40  (Rv2371) | PE_PGRS58  (Rv3590c) | PPE14  (Rv0915c) | PPE32  (Rv1808) | PPE52  (Rv3144c) | PPE69  (Rv3892c) |
| PE3  (Rv0159c) | PE23  (Rv2328) | PE_PGRS6  (Rv0532) | PE_PGRS24  (Rv1325c) | PE_PGRS42  (Rv2487c) | PE_PGRS59  (Rv3595c) | PPE15  (Rv1039c) | PPE33  (Rv1809) | PPE53  (Rv3159c) | PPE9  (Rv0388c) |
| PE4  (Rv0160c) | PE24  (Rv2408) | PE_PGRS7  (Rv0578c) | PE_PGRS25  (Rv1396c) | PE_PGRS43  (Rv2490c) | PE_PGRS60  (Rv3652) | PPE16  (Rv1135c) | PPE34  (Rv1917c) | PPE54  (Rv3343c) | PPE31  (Rv1807) |
| PE5  (Rv0285) | PE25  (Rv2431c) | PE_PGRS9  (Rv0746) | PE_PGRS26  (Rv1441c) | PE_PGRS44  (Rv2591) | PE_PGRS61  (Rv3653) | PPE17  (Rv1168c) | PPE35  (Rv1918c) | PPE55  (Rv3347c) | PPE40  (Rv2356c) |
| PE6  (Rv0335c) | PE26  (Rv2519) | PE_PGRS10  (Rv0747) | PE_PGRS27  (Rv1450c) | PE_PGRS45  (Rv2615c) | PE_PGRS62  (Rv3812) | PPE18  (Rv1196) | PPE36  (Rv2108) | PPE56  (Rv3350c) | PPE47/PPE48  (Rv3021c) |
| PE7  (Rv0916c) | PE27  (Rv2769c) | PE_PGRS11  (Rv0754) | PE_PGRS28  (Rv1452c) | PE_PGRS46  (Rv2634c) | PPE1  (Rv0096) | PPE19  (Rv1361c) | PPE37  (Rv2123) | PPE57  (Rv3425) | PE10  (Rv1089) |
| PE8  (Rv1040c) | PE29  (Rv3022A) | PE_PGRS12  (Rv0832) | PE_PGRS29  (Rv1468c) | PE_PGRS47  (Rv2741) | PPE2  (Rv0256c) | PPE20  (Rv1387) | PPE38  (Rv2352c) | PPE58  (Rv3426) | PE11  (Rv1069c) |
| PE9  (Rv1088) | PE31  (Rv3477) | PE_PGRS13  (Rv0833) | PE_PGRS30  (Rv1651c) | PE_PGRS48  (Rv2853) | PPE3  (Rv0280) | PPE21  (Rv1548c) | PPE39  (Rv2353c) | PPE59  (Rv3429) | PE21  (Rv2099c#) |
| PE12  (Rv1172c) | PE32  (Rv3622c) | PE_PGRS14  (Rv0834c) | wag22  (Rv1759c) | PE_PGRS49  (Rv3344c) | PPE4  (Rv0286) | PPE22  (Rv1705c) | PPE41  (Rv2430c) | PPE60  (Rv3478) | PE28  (Rv3020c) |
| PE13  (Rv1195) | PE33  (Rv3650) | PE_PGRS15  (Rv0872c) | PE_PGRS31  (Rv1768) | PE_PGRS50  (Rv3345c) | PPE5  (Rv0304c) | PPE23  (Rv1705c) | PPE42  (Rv2608) | PPE61  (Rv3532) | LipY  (Rv3097c) |
| PE14  (Rv1214c) | PE34  (Rv3746c) | PE_PGRS16  (Rv0977) | PE_PGRS32  (Rv1803c) | PE_PGRS51  (Rv3367) | PPE6  (Rv0305c) | PPE24  (Rv1753c) | PPE43  (Rv2768c) | PPE62  (Rv3533c) | PE_PGRS8  (Rv0742) |
| PE15  (Rv1386) | PE35  (Rv3872) | PE_PGRS17  (Rv0978c) | PE_PGRS33  (Rv1818c) | PE_PGRS52  (Rv3388) | PPE7  (Rv0354c) | PPE25  (Rv1787) | PPE44  (Rv2770c) | PPE63  (Rv3539) | PE_PGRS36  (Rv2098c) |
| PE16  (Rv1430) | PE36  (Rv3893c) | PE_PGRS18  (Rv0980c) | PE_PGRS34  (Rv1840c) | PE_PGRS53  (Rv3507) | PPE8  (Rv0355c) | PPE26  (Rv1789) | PPE45  (Rv2892c) | PPE64  (Rv3558) | PE_PGRS41  (Rv2396) |
| PE17  (Rv1646) | PE_PGRS1  (Rv0109) | PE_PGRS19  (Rv1067c) | PE_PGRS35  (Rv1983) | PE_PGRS54  (Rv3508) | PPE10  (Rv0442c) | PPE27  (Rv1790) | PPE46  (Rv3018c) | PPE65  (Rv3621c) | |
| PE18  (Rv1788) | PE_PGRS2  (Rv0124) | PE_PGRS20  (Rv1068c) | PE_PGRS37  (Rv2126c) | PE_PGRS55  (Rv3511) | PPE11  (Rv0453) | PPE28  (Rv1800) | PPE49  (Rv3125c) | PPE66  (Rv3738c) | |
| PE19(Rv1791) | PE_PGRS3  (Rv0278c) | PE_PGRS21  (Rv1087) | PE_PGRS38  (Rv2162c) | PE_PGRS56  (Rv3512) | PPE12  (Rv0755c) | PPE29  (Rv1801) | PPE50  (Rv3135) | PPE67  (Rv3739c) | |

^a^The letters in the parentheses indicates the corresponding gene ID in H37Rv (NC_000962).

**Table S5.The information of the Illumina Hiseq reads used for genome sequence correction.**

|  | **Clean reads** | **Reads after duplication removed** | **Quality filtered reads** | **Mate pair reads** | **Mapped reads** | **Coverage** | **Insertions/Deletions resovled** |
| --- | --- | --- | --- | --- | --- | --- | --- |
| Mtb F1 | 3007398*2 | 3004310*2 | 2971281/2927923 | 2906465*2 | 5,774,184(99.33%) | 162 | 438 |
| Mtb F28 | 4379423*2 | 4373320*2 | 4347345/4252729 | 4240596*2 | 8,377,112(98.77%) | 235 | 103 |
| Mtb 22115 | 4395240*2 | 4389699*2 | 4359668/4292518 | 4276688*2 | 8,447,068(98.76%) | 238 | 270 |
| Mtb 37004 | 4549730*2 | 4526196*2 | 4487682/4389369 | 4369689*2 | 8,630,797(98.76%) | 242 | 412 |
| Mtb 22103 | 4520328*2 | 4512583*2 | 4486994/4403619 | 4391167*2 | 8,645,290(98.44%) | 244 | 215 |
| Mtb 26105 | 4115526*2 | 4107048*2 | 4084427/4005086 | 3995648*2 | 7,784,698(97.41%) | 219 | 237 |
| Mtb 2242 | 11093382*2 | 10332899*2 | 9567323/9176825 | 8633342*2 | 17,167,848(99.43%) | 379 | 924 |
| Mtb 2279 | 11303281*2 | 10324255*2 | 9778972/9406245 | 8959324*2 | 17,784,651(99.25%) | 394 | 1392 |
| *M. africanum* 25 | 4192150*2 | 4184931*2 | 4160063/4080122 | 4068029*2 | 8,036,794(98.78%) | 227 | 2119 |
| *M. microti* 12 | 3868797*2 | 3864835*2 | 3850608/3737064 | 3730572*2 | 7,416,883(99.41%) | 211 | 767 |
| *M. bovis* 30 | 2880324*2 | 2873642*2 | 2843805/2815674 | 2794860*2 | 5,492,234(98.26%) | 157 | 471 |
| *M.bovis* BCG 26 | 6423932*2 | 6409310*2 | 6364747/6288711 | 6263596*2 | 12,432,318(99.24%) | 355 | 2278 |

**Table S6.** ANI analysis between human and animal isolates.

|  | ***M. microti* 12** | ***M. bovis* 30** | ***M. bovis* BCG 26** |  |
| --- | --- | --- | --- | --- |
| **Mtb F1** | 99.78% | 99.77% | 99.76% | Average ANI  99.76% |
| **Mtb F28** | 99.77% | 99.76% | 99.74% |  |
| **Mtb 22115** | 99.76% | 99.76% | 99.73% |  |
| **Mtb 37004** | 99.78% | 99.78% | 99.75% |  |
| **Mtb 22103** | 99.76% | 99.74% | 99.71% |  |
| **Mtb 26105** | 99.80% | 99.78% | 99.77% |  |
| **Mtb 2242** | 99.77% | 99.76% | 99.72% |  |
| **Mtb 2279** | 99.77% | 99.75 % | 99.74% |  |

**Table S7.** Animal-associated strains specific genes.

| **Gene^a^** | **Synonym** | **Product_length** | **COG** | **Annotation** |
| --- | --- | --- | --- | --- |
| **-** | Mbo30_144 | 111 | - | hypothetical protein |
| **-** | Mbo30_173 | 52 | - | hypothetical protein |
| **-** | Mbo30_258 | 155 | - | hypothetical protein |
| **-** | Mbo30_883 | 184 | COG3573R | Fumarate/succinate/L-aspartate dehydrogenases |
| **-** | Mbo30_1024 | 49 | - | hypothetical protein |
| **mmpS6** | Mbo30_1740 | 148 | - | membrane protein MmpS family |
| **-** | Mbo30_1981 | 624 | - | PE-PGRS family protein |
| **esxM** | Mbo30_2005 | 98 | COG4842S | ESAT-6-like protein |
| **-** | Mbo30_2079 | 77 | - | hypothetical protein |
| **-** | Mbo30_2097 | 108 | - | hypothetical protein |
| **cobLa** | Mbo30_2318 | 62 | COG2241H | Cobalt-precorrin-6y C5-methyltransferase |
| **-** | Mbo30_3018 | 44 | - | PE family protein |
| **-** | Mbo30_3268 | 366 | COG1819GC | glycosyl transferase family protein |
| **-** | Mbo30_3386 | 60 | - | hypothetical protein |
| **-** | Mbo30_3754 | 51 | COG0399M | hypothetical protein |
| **cyp142b** | Mbo30_3875 | 195 | COG2124Q | cytochrome P450 monooxygenase |

^a^Reference genome: *M. bovis* 30 (CP010332).

**Table S8.** SNPs between human and animal isolates.

**Table S9.** The information of some published complete MTBC genomes used in this study.

A. The information of ten complete genomes of Beijing strains.

| **Organism/Name** | **Strain** | **Size (Mb)** | **GC%** | **Accession Number** | **Genes** |
| --- | --- | --- | --- | --- | --- |
| *M. tuberculosis* CCDC5180 | CCDC5180 | 4.40598 | 65.6 | NC_017522.1/CP001642.1 | 4131 |
| *M. tuberculosis* CCDC5079 | CCDC5079 | 4.41432 | 65.6 | NC_021251.1/CP002884.1 | 4136 |
| *M. tuberculosis* HKBS1 | HKBS1 | 4.40793 | 65.6 | NZ_CP002871.1/CP002871.1 | 4126 |
| *M. tuberculosis* BT2 | BT2 | 4.4019 | 65.6 | NZ_CP002882.1/CP002882.1 | 4122 |
| *M. tuberculosis* BT1 | BT1 | 4.3994 | 65.6 | NZ_CP002883.1/CP002883.1 | 4125 |
| *M. tuberculosis* K | K | 4.38552 | 65.6 | NZ_CP007803.1/CP007803.1 | 4102 |
| *M. tuberculosis* | KIT87190 | 4.41079 | 65.6 | NZ_CP007809.1/CP007809.1 | 4125 |
| *M. tuberculosis* | 96075 | 4.37938 | 65.6 | NZ_CP009426.1/CP009426.1 | 4114 |
| *M. tuberculosis* 49-02 | 17930 | 4.41238 | 65.6 | NZ_HG813240.1/HG813240.1 | 4126 |
| *M. tuberculosis* | Beijing-like | 4.41122 | 65.6 | CP010873.1 | 4170 |

B. The information of five *M. tuberculosis* H37Rv and two *M. tuberculosis* H37Ra complete genomes.

| **Organism/Name** | **Strain** | **Size (Mb)** | **GC%** | **Accession Number** | **Genes** |
| --- | --- | --- | --- | --- | --- |
| *M. tuberculosis* H37Rv | H37Rv | 4.41 | 65.6 | NC_000962.3/AL123456.3 | 4008 |
| *M. tuberculosis* H37Rv | H37Rv | 4.41 | 65.6 | NC_018143.2/CP003248.2 | 4132 |
| *M. tuberculosis* H37RvSiena | H37RvSiena | 4.41 | 65.6 | CP007027.1 | 4133 |
| *M. tuberculosis* H37Rv | H37Rv; TMC 102 | 4.40 | 65.6 | CP009480.1 | 4135 |
| *M. tuberculosis* H37Rv | F1 | 4.40 | 65.6 | CP010329.1 | 4400 |
| *M. tuberculosis* H37Ra | H37Ra; ATCC 25177 | 4.42 | 65.6 | NC_009525.1/CP000611.1 | 4153 |
| *M. tuberculosis* H37Ra | F28 | 4.40 | 65.6 | CP010330.1 | 4366 |

C. The information of three *M. bovis* and nine *M. bovis* BCG complete genomes.

| **Organism/Name** | **Strain** | **Size (Mb)** | **GC%** | **Accession Number** | **Genes** |
| --- | --- | --- | --- | --- | --- |
| *M. bovis* | AF2122/97 | 4.35 | 65.6 | NC_002945.3/BX248333.1 | 4001 |
| *M. bovis* | 1595 | 4.35 | 65.6 | CP012095.1 | 4061 |
| *M. bovis* | 30 | 4.34 | 65.6 | CP010332.1 | 4312 |
| *M. bovis* BCG | BCG Pasteur 1173P2 | 4.37 | 65.6 | NC_008769.1/AM408590.1 | 4079 |
| *M. bovis* BCG | Tokyo 172 | 4.37 | 65.6 | NC_012207.1/AP010918.1 | 4080 |
| *M. bovis* BCG | 63839 | 4.33 | 65.7 | CP003494.1 | 4055 |
| *M. bovis* BCG | Mexico | 4.35 | 65.7 | NC_016804.1/CP002095.1 | 4052 |
| *M. bovis* BCG | Korea 1168P | 4.38 | 65.6 | NC_020245.2/CP003900.2 | 4080 |
| *M. bovis* BCG | Moreau RDJ | 4.34 | 65.6 | NZ_AM412059.1/AM412059.2 | 4054 |
| *M. bovis* BCG | 3281 | 4.41 | 65.6 | CP008744.1 | 4107 |
| *M. bovis* BCG | Russia 368 | 4.37 | 65.6 | CP009243.1 | 4079 |
| *M. bovis* BCG | 26 | 4.35 | 65.6 | CP010331.1 | 4348 |

**Table S10.** *M. bovis* and *M. bovis* BCG specific genes

A. *M. bovis* specific genes.

| **Gene**^a^ | **Synonym** | **Product accession** | **Product length** | **COG** | **Annotation** |
| --- | --- | --- | --- | --- | --- |
| **PE_PGRS20** | Mb1097c | NP_854752.1 | 763 | COG4907S | PE-PGRS family protein |
| **-** | Mb1188c | NP_854843.1 | 371 | COG5373S | hypothetical protein |
| **treYa** | Mb1590c | NP_855242.1 | 250 | COG3280G | malto-oligosyltrehalose synthase |
| **-** | Mb1599 | NP_855252.1 | 136 | - | phiRV1 phage protein |
| **-** | Mb1600 | NP_855253.1 | 103 | - | phiRV1 phage-like protein |
| **-** | Mb1602c | NP_855255.1 | 473 | COG4653R | phiRV1 phage protein |
| **-** | Mb1603c | NP_855256.1 | 170 | COG3740R | phiRv1 phage protein |
| **-** | Mb1604c | NP_855257.1 | 156 | COG3747L | phiRv1 phage protein |
| **-** | Mb1605c | NP_855258.1 | 104 | - | phiRv1 phage protein |
| **-** | Mb1606c | NP_855259.1 | 90 | - | phiRv1 phage protein |
| **-** | Mb1607c | NP_855260.1 | 131 | - | phiRv1 phage protein |
| **-** | Mb1608c | NP_855261.1 | 471 | COG3378R | phiRv1 phage protein |
| **-** | Mb1609c | NP_855262.1 | 132 | - | phiRv1 phage protein |
| **-** | Mb1610c | NP_855263.1 | 73 | - | phiRv1 phage protein |
| **-** | Mb1611c | NP_855264.1 | 171 | - | phiRv1 phage protein |
| **-** | Mb1612c | NP_855265.1 | 469 | COG1961L | phiRv1 integrase |
| **PPE40** | Mb2377c | NP_856026.1 | 614 | COG5651N | PPE family protein |
| **ugpAa** | Mb2860c | NP_856505.1 | 123 | COG1175G | sn-glycerol-3-phosphate ABC transporter integral membrane protein |
| **fadE22a** | Mb3088c | NP_856733.1 | 114 | COG1960I | acyl-CoA dehydrogenase |
| **PE35** | Mb3902 | NP_857539.1 | 98 | - | PE family protein PE35 |
| **PPE68** | Mb3903 | NP_857540.1 | 368 | COG5651N | PPE family protein PPE68 |
| **esxB** | Mb3904 | NP_857541.1 | 100 | COG4842S | ESAT-6-like protein EsxB |
| **esxA** | Mb3905 | NP_857542.1 | 95 | COG4842S | ESAT-6 protein EsxA |
| **espI** | Mb3906 | NP_857543.1 | 666 | COG0455D | ESX-1 secretion-associated protein EspI |
| **eccD1** | Mb3907 | NP_857544.1 | 511 | - | ESX-1 secretion system protein EccD1 |
| **espJ** | Mb3908 | NP_857545.1 | 280 | - | ESX-1 secretion-associated protein EspJ |
| **espK** | Mb3909c | NP_857546.1 | 744 | COG3225N | ESX-1 secretion-associated protein EspK |
| **-** | Mb0074 | NP_853743.1 | 280 | COG1136V | glutamine-transport ATP-binding protein ABC transporter GlnQ |

^a^Reference genome: *M. bovis* (NC_002945).

B. *M. bovis* BCG specific genes.

| **Gene^a^** | **Synonym** | **Product accession** | **Product length** | **COG** | **Annotation** |
| --- | --- | --- | --- | --- | --- |
| **-** | BCG_1218c | CAL71205.1 | 347 | COG0508C | Conserved hypothetical ala-, pro-rich protein |
| **treZb** | BCG_1614c | CAL71601.1 | 186 | COG0296G | Maltooligosyltrehalose trehalohydrolase TreZ |
| **-** | BCG_2624 | CAL72612.1 | 59 | - | Probable conserved integral membrane protein |
| **-** | BCG_0315A | CAL70300.1 | 85 | - | hypothetical protein, antitoxin |
| **PPE49b** | BCG_3146c | CAL73135.1 | 104 | - | PPE family protein |
| **-** | BCG_3943c | CAL73933.1 | 220 | COG0455D | Conserved membrane protein |

^a^Reference genome: *M. bovis* BCG (NC_008769).

**Table S11.** SNPs between *M.bovis* and *M.bovis* BCG.

**Table S12.** Detailed information of MTBC epitopes used in this paper.

**Table S13**. Lost epitopes in *M. bovis* BCG 26 and *M. microti* 12.

**Table S14.** The genetic variations on antigen genes among the 12 MTBC strains.

| Gene Name | Gene ID in NC_000962 | Position^a^ | Variation^b^ | L4 | | | | | L3 | L2 | | L6 | L8 | | | Lost Epitopes |
| --- | --- | --- | --- | --- | --- | --- | --- | --- | --- | --- | --- | --- | --- | --- | --- | --- |
|  |  |  |  | MtbF1 | Mtb  F28 | Mtb  22115 | Mtb  37004 | Mtb  22103 | Mtb  26105 | Mtb  2242 | Mtb  2279 | *M.*  *africanum*  25 | *M.microti*  12 | *M.bovis*  30 | *M.bovis*  BCG 26 |  |
| vapB17 | Rv2526 | 71 | AAACATT | - | - | - | - | - | - | AC | AC | - | - | - | - | Tcell_0579, 0591 |
| ccsA | Rv0529 | 735(245) | A(I) | - | - | - | - | - | - | G (M) | G(M) | - | - | - | - | Tcell_1737 |
| - | Rv2628 | 176(59) | C(S) | - | - | - | - | - | - | - | - | T(L) | T(L) | T(L) | T(L) | Tcell_1033 |
| PE12 | Rv1172c | 399(133) | A(Q) | - | - | - | - | - | - | - | - | C(H) | C(H) | C(H) | C(H) | Tcell_1623 |
| mmpL8 | Rv3823c | 703(235) | G(G) | - | - | - | - | - | - | - | - | C(R) | C(R) | C(R) | C(R) | Tcell_1612 |
| esxO | Rv2346c | 65(22) | G(G) | - | - | - | - | - | - | - | C(A) | C(A) | C(A) | C(A) | C(A) | Tcell_0247 |
|  |  | 68(23) | T(L) | - | - | - | - | - | - | - | C(S) | C(S) | C(S) | C(S) | C(S) |  |
| - | Rv1877 | 705 | C | - | - | - | - | - | - | - | - | - | CC | - | - | Tcell_1581, 1611 |
|  |  | 801 | C | - | - | - | - | - | - | - | - | - | - | CC | CC |  |
| PPE46 | Rv3018c | 149(50) | T(V) | - | - | - | - | - | - | - | - | C(A) | C(A) | C(A) | C(A) | Tcell_1622 |
| gnd1 | Rv1844c | 325(109) | - | - | - | - | - | - | - | - | - | - | InsATC (InsI) | / | / | Tcell_1718 |
| - | Rv2659c | 294(98) | C(H) | - | - | - | - | - | - | - | - | / | G(Q) | / | / | Tcell_0181, Tcell_0183 |
| pstS1 | Rv0934 | 1055(352) | T(V) | - | - | - | - | - | - | - | - | C(A) | C(A) | C(A) | C(A) | Tcell_0007,0627,0657,2149,2150; Bcell_0006,  0440,0453 |
| pstS2 | Rv0932c | 343(115) | T(S) | - | - | - | - | - | - | - | - | G(A) | G(A) | G(A) | G(A) | Bcell_00419,  0455 |
| - | Rv2958c | 868 | G | - | - | - | - | - | - | - | - | GG | GG | GG | GG | Bcell_0439,  0485 |

^a^The number in the parentheses indicates the mutant position in protein;

^b^The letters in the parentheses indicates the amino acid substitutions;

"/": Gene deletion；"-": No variation; "Ins": Insertion.

**Table S15.** Variations between *M.bovis* and *M.bovis* BCG on antigens.

|  | **Gene Name** | **GI** | **Position** | **AA^a^** | ***M.bovis*** | ***M.bovis*BCG** |
| --- | --- | --- | --- | --- | --- | --- |
| **Tcell/Bcell** | fbpB | 398979/158517767/148661692 | 140 | F | - | L |
| **Tcell** | mce4C | 15610633 | 46 | T | - | P |
|  | PPE19 | 148661151 | 325 | Q | - | P |
|  | alpha-antigen | 149936/44168/121493437 | 140 | L | F | - |
|  | PPE18 | 124600519 | 122 | I | - | T |
|  | PPE27 | 1092883 | 190 | S | - | P |
|  | eccCb | 15843502 | 480-581 |  | - | Indels |

^a^Amino Acid.

**REFERENCE**

Zhu, L., Zhong, J., Jia, X., Liu, G., Kang, Y., Dong, M., et al. (2016). Precision methylome characterization of *Mycobacterium tuberculosis* complex (MTBC) using PacBio single-molecule real-time (SMRT) technology. *Nucleic Acids Res*. 44, 730–743. doi: 10.1093/nar/gkv1498

Zhao, Y., Xu, S., Wang, L., Chin, D., Wang, S., Jiang, G., et al. (2012). National Survey of Drug-Resistant Tuberculosis in China. *N. Engl. J. Med.* 366(23), 2161-2170. doi: 10.1056/NEJMoa1108789
